# Curiosity and surprise differentially affect memory depending on age

**DOI:** 10.1038/s41598-025-14479-x

**Published:** 2025-09-12

**Authors:** Alexandra Sobczak, Tineke Steiger, Marthe Mieling, Nico Bunzeck

**Affiliations:** 1 Department of Psychology, University of Lübeck, Maria-Goeppert-Strasse 9a, 23562 Lübeck, Germany; 2 Center of Brain, Behavior and Metabolism, University of Lübeck, Lübeck, Germany

**Keywords:** Curiosity, Surprise, Education, Aging, Long-term memory, Human behaviour, Cognitive ageing

## Abstract

State and trait epistemic curiosity can promote various aspects of cognition and behavior. However, possible age-related changes, a link to surprise, and formal education remain understudied. In three behavioral experiments, with *n* = 54 in experiment 1, *n* = 81 in experiment 2, and *n* = 196 in experiment 3, we could show that state but not trait epistemic curiosity drives long-term memory for relevant information in young and healthy older adults (experiment 1 and 2). This effect was further modulated by surprise in young but less in older adults, which is compatible with predictive coding theories and the notion of a quadratic effect of arousal on cognition that vanishes with age (experiment 2). Finally, in a mediation model, state epistemic curiosity could be predicted directly from trait epistemic curiosity and indirectly via formal education (experiment 3). Our findings specify how state and trait curiosity, together with surprise, promote learning and facilitate knowledge acquisition across the adult lifespan.

**Supplementary Information:**

The online version contains supplementary material available at 10.1038/s41598-025-14479-x.

## Introduction

Curiosity is a powerful form of intrinsic motivation enabling us to seek and explore novel information, thereby shaping individual development, including educational achievement. While many studies, that will be explained below, gave valuable insights, the lifelong developmental trajectories and possible age-related changes remain unclear^1,2^. Addressing this important gap may not only have practical implications with regard to healthy aging but it could also advance our conceptual understanding and theoretical models^3–5^. Here, we specifically focus on epistemic curiosity (EC), i.e. the drive to acquire new knowledge in order to close an “information gap”^6^. EC can further be distinguished into state and trait EC, which relates to a rather stable and consistent desire to acquire new knowledge (i.e. trait EC), or a transient and situational intrinsic motivation to acquire new knowledge (i.e. state EC)^6–8^.

The ability to learn and encode novel information into long-term memory (LTM) strongly depends on different types of motivation. Studies from both psychology and cognitive neuroscience have shown that monetary rewards (i.e. extrinsic motivation) and state EC (i.e. intrinsic motivation) promote learning and memory performance via the dopaminergic mesolimbic system and interconnected brain regions. For instance, superior memory performance is being observed when images serve as cues for monetary feedback^9,10 ^and these behavioral effects were linked to hemodynamic activity in the substantia nigra / ventral tegmental area (SN/VTA), nucleus accumbens and hippocampus^11^. Similarly, being in a state of high curiosity, that can be induced by unknown answers to trivia questions^12,13^ or magic tricks^14,15^, leads to enhanced mesolimbic activity and promotes subsequent memory formation not only for the curios item but also for incidental information. From a conceptual point of view, the positive effects of curiosity (and reward) on LTM can be explained by predictive coding theories^16,17^ and studies demonstrating that “information prediction errors” (i.e. differences in perception and prediction) can enhance learning^18^. Therefore, intrinsic (i.e. curiosity) and extrinsic (i.e. money) types of motivation modulate declarative learning via the mesolimbic system, which points towards common psychological and neurobiological underpinnings.

This latter notion is important since the mesolimbic system typically degenerates during healthy aging with functional consequences for reward processing and the ability to acquire novel information. For instance, age-related degeneration of the dopaminergic midbrain affects neural novelty signals^19^ and LTM performance^20^, while iron levels and myelin content in the ventral striatum predict memory performance in the aging brain^21^. Moreover, reward anticipation relates to neural theta oscillations (i.e. 4–8 Hz) and is predicted by dopaminergic midbrain integrity in healthy older adults^22^. In line with this observation, healthy older adults did not show the typical reward anticipation signal in the mesolimbic system^23^, suggesting that impairments in motivational processing can be explained by region specific degenerations. However, the positive effect of state EC on learning and memory performance in healthy older adults appears *not* to be impaired as compared to their younger counterparts. In fact, two initial studies showed that state EC increased subsequent LTM in both young and older humans with no significant differences between both age groups^24,25^. While this suggests that learning in older adults still benefits from state EC, it remains unclear how this relates to the previously described degeneration of the mesolimbic system and typical age-related declarative memory impairments^26^.

**Fig. 1:**
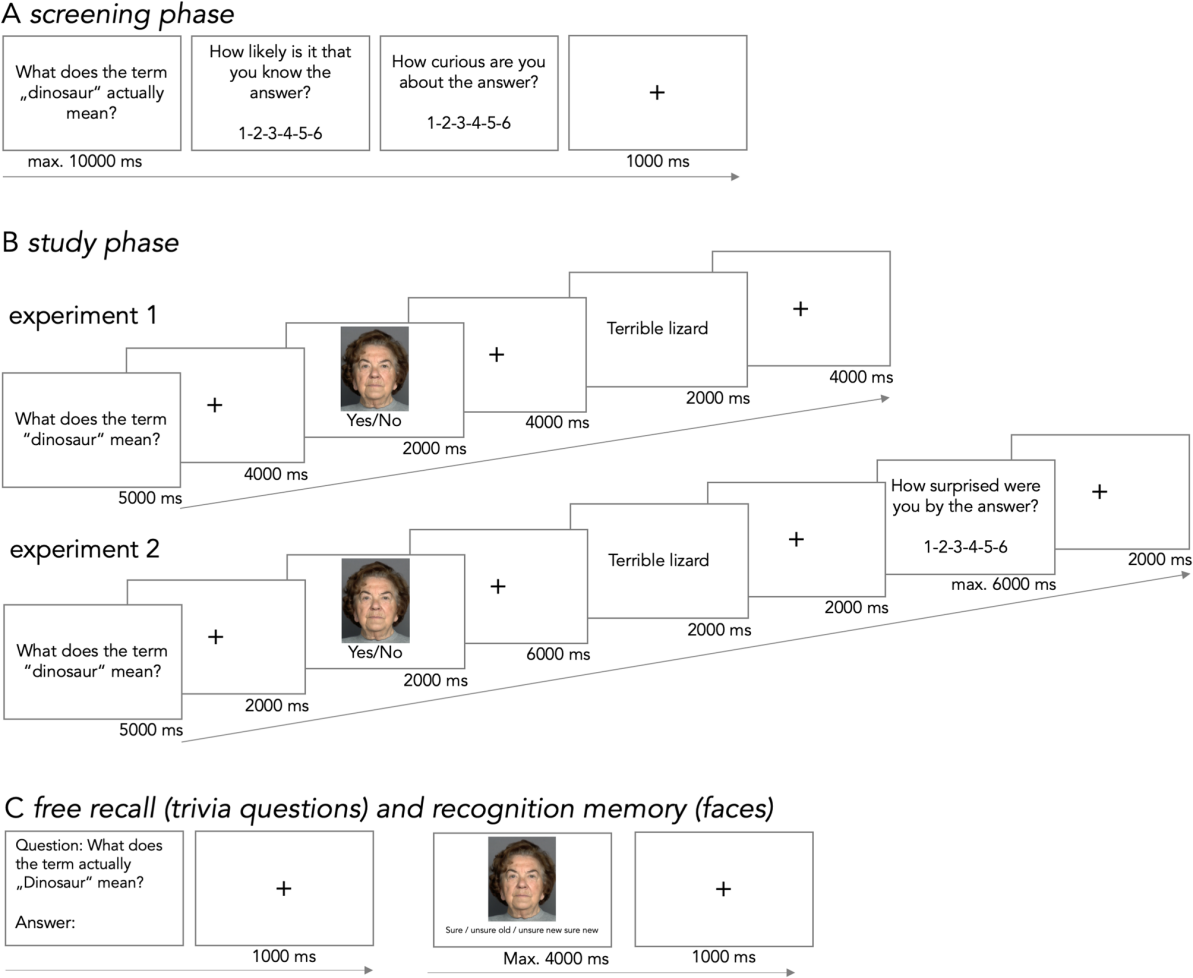
Study design for experiment 1 and 2. Both experiments included a screening phase (**A**) and a study phase (**B**) on day one as well as a free recall and recognition memory test on the subsequent day (**C**). In the screening phase (**A**) subjects rated their knowledge and curiosity to trivia questions. In the study phase (**B**), the trivia questions were presented again followed by a trial unique face and the correct answer to the question. In experiment 2, subjects additionally indicated how surprised they were by the answer to each trivia question. The memory test (**C**) included a free recall of the answers to the trivia questions and a recognition memory test for the faces. All presented faces (also shown here) were taken from the “FACES” database^56^. See text for more information.

One possibility is that other factors – in particular surprise about the answer to the curious question – play an important role. This hypothesis is based on predictive coding theories^16,17^, suggesting a close relationship of novelty encoding, surprise and prediction errors^27–30^. In fact, prediction errors are typically reduced in older adults possibly due to more accurate generative models of the world (due to larger semantic networks) or the inability to properly detect deviations (due to cognitive impairments)^31^. In contrast, sensitivity to surprise appears to be enhanced in older adults as shown in studies on reinforcement learning^32 ^and facial processing^33^. Moreover, a study in young adults indicates that surprise can have a mediating effect on subsequent memory performance via the rostrolateral prefrontal cortex^29^, which is compatible with a recent framework suggesting a close link between prediction errors, appraisal, curiosity, and exploration (PACE)^34^. Therefore, in study 1 (Fig. 1), which was based on previous work^12,25^, we aimed to replicate the absence of an interaction between age and curiosity on LTM performance^24,25^; and in experiment 2 (Fig. 1), we further investigated a possible interaction between age, curiosity and surprise on LTM performance.

In contrast to state EC, trait EC is considered a stable personality characteristic that can be further divided into interest (I) and deprivation (D) type EC^35^. While trait I-EC indicates the intrinsic pleasure of learning, trait D-EC is associated with an intense need to resolve knowledge gaps. Both types have been linked to motivation and performance in academic contexts^36^, including goal setting and achievement^35 ^but also emotional intelligence^37^, intellectual development^38,39^as well as empathetic and research abilities^40^. Moreover, recent research highlights a bidirectional relationship between curiosity and knowledge, suggesting that curiosity not only fosters academic achievement but can also be shaped by prior learning experiences^41,42^. This raises the question of how trait and state curiosity are related to long-term developmental outcomes – most notably, formal educational attainment, which represents a key marker of cognitive engagement. To address this issue, experiment 3 explored the relationship between trait EC, state EC, and formal education, and tested whether educational achievements mediate the link between trait and state EC.

With regard to age-related changes, trait curiosity, especially intellectual curiosity, typically declines with age^43, which is ^consistent with findings from personality research on openness^44,45^. Interestingly, the association between age and intellectual curiosity appears to be mediated by future time perspective^46^, which is compatible with the socioemotional selectivity theory^47,48 ^suggesting that older adults often prioritize emotionally meaningful over exploratory goals. Age-dependent changes in state curiosity, on the other hand, are context-dependent and influenced by subjective aspects. For instance, despite lower levels in trait curiosity, older adults can exhibit higher state curiosity when the interaction with robots was personally relevant^49^.

While experiment 1 and 2 each included two groups of healthy young and older adults in a laboratory setting (Fig. 1), experiment 3 was based on an online survey with subjects across the adult lifespan. We expected a positive effect of state EC on learning and memory of intentional information (i.e. answers to trivia questions) and incidental information (i.e. faces presented in between questions and answers) with no significant differences between age groups replicating previous findings^24,25^, experiment 1. Note that positive effects of curiosity on incidental information are rather weak and inconsistent^12,25 ^and, therefore, require further attention. We also hypothesized a positive effect of surprise on LTM performance^29^ and explored differences between both age groups given the typical structural and functional changes in underlying brain regions^20,26,31^, experiment 2, Finally, we expected that state EC could be predicted based on trait EC and educational attainment, and we further investigated their relationship in a mediation analysis, experiment 3.

## Methods

### Experiment 1

#### Participants

Fifty-nine healthy human subjects participated in experiment 1. Data from 25 young participants (age range 19–30 years, M = 22.4, SD = 3.0; female = 13) and 29 older participants (age range 53–76 years, M = 66.7, SD = 6.45; female = 16) were analyzed, in total *n* = 54. Data of the remaining subjects were discarded due to technical reasons (*n* = 5), or since their performance did not meet the requirements (*n* = 1).

All participants were right-handed, had a normal or corrected-to-normal vision, no history of neurological or psychiatric disorders (self-report), and were fluent in German language. All older participants were screened for mild cognitive impairment (MCI) or dementia using the Montreal Cognitive Assessment (MoCA)^50^ with a cut-off of 22^51^. They were also screened for depression using a German version of the Geriatric Depression Scale (GDS)^52,53^ with a cut-off of 6^52^. Participants were recruited through the Online Recruitment System for Economic Experiments (ORSEE^54)^.

#### Study design and procedure

##### State curiosity and memory performance.

The study design was based on previous work^12,25^ and comprised three phases (Fig. 1): (a) *screening phase*, (b) *study phase*, and (c) final *retrieval phase* (1 day after study). In the screening phase (a), subjects were presented with a series of trivia questions from different domains including “science and nature”, “history”, and “entertainment”. After each question, subjects had to indicate whether they know the answer or not on a Likert scale from 1 to 6 (1: “I am very confident that I do *not* know the answer”; 6: “I am very confident that I know the answer”). Subsequently, they indicated how curious they were to know the answer to the question, again on a Likert scale from 1 to 6 (1: “I am not at all curious to know the answer”; 6: “I am very curious to know the answer”). This self-paced procedure typically took 30–50 min and continued until 56 questions per high and low curiosity category were reached (curiosity scores of 1–3 correspond to a *low curiosity* category and 4–6 to a *high curiosity* category; questions with an initial answer of 6, i.e. subjects are very sure to know the answer, were disregarded). If one of the two categories (high / low curiosity) was filled with more than 56 items, we randomly chose 56 of them for the subsequent study phase. Subjects were not informed that 56 items were necessary to proceed with the experiment.

For the subsequent study phase (b), subjects were presented with all previously collected questions again (i.e. 112 in total, 56 per high and low curiosity category). For each trial (Fig. 1B), a question (5 s) was followed by a 12 s long *anticipation phase* including a trial unique face stimulus after 4 s, and it ended with the answer to the question, again after 4 s. While questions and answers had to be read, the face stimulus required a decision on how knowledgeable the person would be to answer the question (yes/no decision via button press). This ensured that subjects paid attention to the face and allowed us to test the hypothesis that curiosity not only drives memory for related but also incidental information^12,55^. Additionally, in six of the high curiosity trials and in six of the low curiosity trials a “catch” trial was added. In this case the answer to the question was replaced by “XXXXX” and subjects had to press the space bar to receive the correct answer. This helped to ensure that subjects paid attention, but catch trials were removed from further analyses. All faces had a neutral expression and were taken from the “FACES” database^56^. With regard to age, they were either young, middle-aged, or old (equal distribution).

The final retrieval phase (c) was performed on the subsequent day. Here, all 112 face stimuli from the study phase (56 from a high curiosity context and 56 from a low curiosity context) were presented intermixed with 56 novel faces in random order. For each face, subjects had to indicate recognition confidence as quickly and accurately as possible (‘‘confident new,’’ ‘‘unconfident new,’’ ‘‘unconfident old,’’ and ‘‘confident old’’). Subjects had a maximum of 4 s to give their response. Finally, subjects were presented with the same trivia questions from the study phase again (on a computer screen in random order), and they had to write down as many answers as possible within max. 30 min. Trivia questions that had been used as catch trials the day before were not used for this phase.

Data for this experiment were acquired using MATLAB R2015a (The MathWorks, Inc., Natick, MA, USA) in combination with Psychophysics Toolbox Version 3 (PTB-3)^57^.

##### Trait curiosity

The German version of an established questionnaire on trait EC^35^ was used. It consists of a total of 10 questions, with five questions on I-EC and five items on D-EC. Thus, for every subject it provides an overall measure of trait EC as well as an individual measure of I-EC and D-EC.

#### Data analysis

All data were analyzed using jamovi (Version 2.3.21.0, retrieved from https://www.jamovi.org). For answers to the trivia questions, free recall rates were quantified based on the subject’s responses. It was calculated by dividing the number of correctly recalled answers (n) by the number of total possible answers (50 per curiosity category) multiplied by 100 (i.e. n /50*100). The absolute numbers per cell are shown in Table S1, for recall rates see results. For the faces, corrected hit rates (CHR) served as a measure of accuracy during recognition and were calculated by subtracting the false alarm rate (incorrect old responses to new items) from the hit rate (correct old responses to old items). Outliers were excluded if necessary (> 3 SD from the mean); there were no outliers for free recall rates on trivia questions (i.e. for low and high curiosity items), or for the CHR on face stimuli (i.e. CHR for low or high curiosity). For both trivia questions and faces, a separate 2 × 2 ANOVA with the between-subject factor group (young, older), and the within-subject factor curiosity (high, low) was performed, followed by planned post hoc t-tests (Bonferroni corrected for multiple comparisons, two-sided) and supplemented by Bayes statistics^58,59^ where appropriate (using default priors, see results).

### Experiment 2

#### Participants

Ninety-seven healthy human subjects participated in this experiment. Data from 42 young participants (age range 18–30 years, M = 23.3, SD = 3.26; female = 37) and 39 older participants (age range 50–88 years, M = 68.3, SD = 7.82; female = 22) were analyzed, in total *n* = 81. Data of the remaining subjects were discarded due to technical reasons (*n* = 12), concentration problems (*n* = 1) or not meeting the inclusion criteria (i.e. Geriatric Depression Scale > 5, *n* = 1, or MoCA < 22, *n* = 2).

All participants were right-handed, had a normal or corrected-to-normal vision, no history of neurological or psychiatric disorders (self-report), and were fluent in German language. All older participants were screened for mild cognitive impairment (MCI) or dementia using the MoCA^50^ with a cut-off of 22 ^51^. They were also screened for depression using a German version of the GDS^52,53^ with a cut-off of 6 ^52^. Participants were recruited through the Online Recruitment System for Economic Experiments (ORSEE^54^).

#### Study design

##### State curiosity and memory performance

The study design was identical to experiment 1 with one exception: in the study phase, after being presented with the answer to the trivial question, all subjects had to indicate their level of surprise on a Likert scale from 1 to 6 (1: “I am not very surprised about the answer”; 6: “I am very surprised about the answer”, Fig. 1B).

Data for this experiment were acquired using MATLAB R2018b (The MathWorks, Inc., Natick, MA, USA) in combination with Psychophysics Toolbox Version 3 (PTB-3)^57^.

##### Trait curiosity

The German version of an established questionnaire on trait EC^35^ was used, allowing us to quantify I-EC and D-EC (see Methods experiment 1).

#### Data analysis

All data were analyzed using jamovi (Version 2.3.21.0, retrieved from https://www.jamovi.org). For answers to the trivia questions, free recall rates were quantified based on the subject’s responses. It was calculated by dividing the number of correctly recalled answers (n) by the number of total possible answers (50 per curiosity category) multiplied by 100 (i.e. n /50*100). The absolute numbers per cell are shown in Table S1, for recall rates see results. For the faces, corrected hit rates (CHR) served as a measure of accuracy during recognition and were calculated by subtracting the false alarm rate (incorrect old responses to new items) from the hit rate (correct old responses to old items). Outliers were excluded if necessary (> 3 SD from the mean); there were no outliers for the trivia questions, but for face stimuli, 15 subjects had to be excluded since they exhibited negative CHR or only “unsure” responses. Therefore, only *n* = 66 subjects remained for the face analysis.

For both trivia questions and faces, a separate 2 × 2 × 2 ANOVA with the between-subject factor group (young, older), and the within-subject factors curiosity (high, low) and surprise (high, low) was performed, followed by planned post hoc t-tests (Bonferroni corrected for multiple comparisons, two-sided) and supplemented by Bayes statistics^58,59^ where appropriate (using default priors, see results).

We also performed a logistic mediation analysis with state EC as predictor, surprise as mediator and recall rate as dependent variable. The analysis was performed in two steps. First, the logistic mediation analysis was calculated on the subject level, resulting in 4 mediation coefficients reflecting (i) the direct effect of state EC on memory, (ii) the direct effect of state EC on surprise, (iii) the direct effect of surprise on memory, and (iv) the indirect effect of state EC on memory mediated by surprise. Second, we tested the significance of each of the four coefficients using one-sample t-tests (two-sided). Here, two subjects had to be excluded since their mediation coefficients deviated more than 3 SD from the group mean. Note, that the first step was based on the PROCESS macro version 4.2 for IBM SPSS statistics (version 29)^60^.

### Experiment 3

#### Participants

231 healthy human subjects participated in an online experiment using SoSci Survey (https://www.soscisurvey.de). Data from *n* = 196 participants (age range 19–82 years, M = 37.3, SD = 15.8; female = 131, male = 59, diverse = 1, not specified = 5) were analyzed. Data of the remaining subjects (*n* = 34) were discarded due to technical reasons, incomplete datasets or missing catch-trials (see below).

#### Study design and procedure

##### State curiosity

All subjects were presented with a state EC questionnaire, including 21 trivia questions from different domains including “science and nature”, “history”, and “entertainment”. After each question, subjects had to indicate whether they know the answer or not on a Likert scale from 1 to 6 (1: “I am very confident that I do *not* know the answer”; 6: “I am very confident that I know the answer”). Subsequently, they indicated how curious they were to know the answer to the question, again on a Likert scale from 1 to 6 (1: “I am not at all curious to know the answer”; 6: “I am very curious to know the answer”). The average curiosity values for unknown questions were used as marker of state EC. Additionally, we included four catch-trials, in which the keyboard button number 4 had to be pressed. Missing a catch-trial resulted in data exclusion (*n* = 23). Note that the number of trials per subject differs compared to experiment 1 and 2 since here, in experiment 3, we did not investigate the direct effect of state EC on subsequent memory (which requires more trials per state curiosity condition). Moreover, online experiments require a trade-off between number of stimuli and length of the experiment (see discussion).

##### Trait curiosity

Similar to experiment 1 and 2, the German version of an established questionnaire on trait EC^35^ was used, allowing us to quantify I-EC and D-EC (see methods experiment 1 and 2).

##### Sociodemographic information

All subjects were asked for their age, gender and highest educational degree (according to the German system). This included: no degree, main school (Hauptschule), secondary school (Realschule), apprenticeship (abgeschlossene Ausbildung), advanced technical college entrance qualification (Fachabitur), German high school diploma (Abitur), degree from an institution of higher education (including University and University of Applied Sciences), and still pupil at school (*n* = 2, who were excluded from the analysis). In our final sample (excluding outliers and those cases mentioned above), 1.5% had no degree (*n* = 3), 1.5% had a main school degree (*n* = 3), 11.8% had a secondary school degree (*n* = 23), 19.5% had an apprenticeship degree (*n* = 38), 8.2% had an advanced technical college entrance qualification (*n* = 16), 25.1% had a German high school diploma (*n* = 49), and 32.3% had a degree from an institution of higher education (*n* = 63). In order to analyze the data with multiple regression and mediation analysis, the information of the highest degree was transformed into an “education score”; accordingly, no degree corresponded to 0 points, main school corresponded to 1, secondary school corresponded to 2, completed apprenticeship and advanced technical college entrance qualification corresponded to 3 (both degrees qualify for a degree at universities), German high school diploma corresponded to 4, and a degree from higher education corresponded to 5 points. Although “education score” was not normally distributed (Shapiro-Wilk-Test, W = 0.87, *p* < 0.001), it did not include any outliers (SD > 3 from the mean), and multicollinearity, linearity and homoscedasticity assumptions were not violated.

We preferred these categorical variables over GPA (Grade Point Average) or similar systems used in other countries, as these are not universally applicable, particularly in the German system. While most German degrees include a final grade, these are not necessarily comparable due to differences in requirements and grading approaches. Moreover, asking participants to recall their highest degree is generally more feasible than recalling GPA, the final grade associated with a degree, or years of education – all of which have their own limitations.

#### Data analysis

All data were analyzed using jamovi (Version 2.3.21.0, retrieved from https://www.jamovi.org). Outliers were excluded if necessary (> 3 SD from the mean). Subsequently, data were analyzed using correlation and mediation analyses (two-sided).

#### Power calculation

The sample sizes are comparable to previous studies on trait and state curiosity (e.g. Chu and Fung, 2022; Galli et al., 2017; Kang et al., 2009; McGillivray et al., 2015). A power calculation (G*Power 3.1) for experiment 1 (ANOVA, 2 groups, 2 measurements) with an effect size of f = 0.25, α = 0.05 and 1-β = 0.95 revealed a total sample size of 54. A power calculation (G*Power 3.1) for experiment 2 (ANOVA, 2 groups, 3 measurements) with an effect size of f = 0.25, α = 0.05 and 1-β = 0.95 revealed a total sample size of 44 (for any possible interaction). For experiment 3, especially the mediation analysis, we aimed for a sample size of at least *n* = 150 based on literature recommendations^61,62^. In all three studies, we used larger sample sizes than suggested to account for potentially smaller effect sizes, unknown variability, attrition, and the need for robust, generalizable, and replicable findings, including minimizing the risks of type I and type II errors.

## Results

### Experiment 1

#### Recall performance on trivia questions

**Fig. 2:**
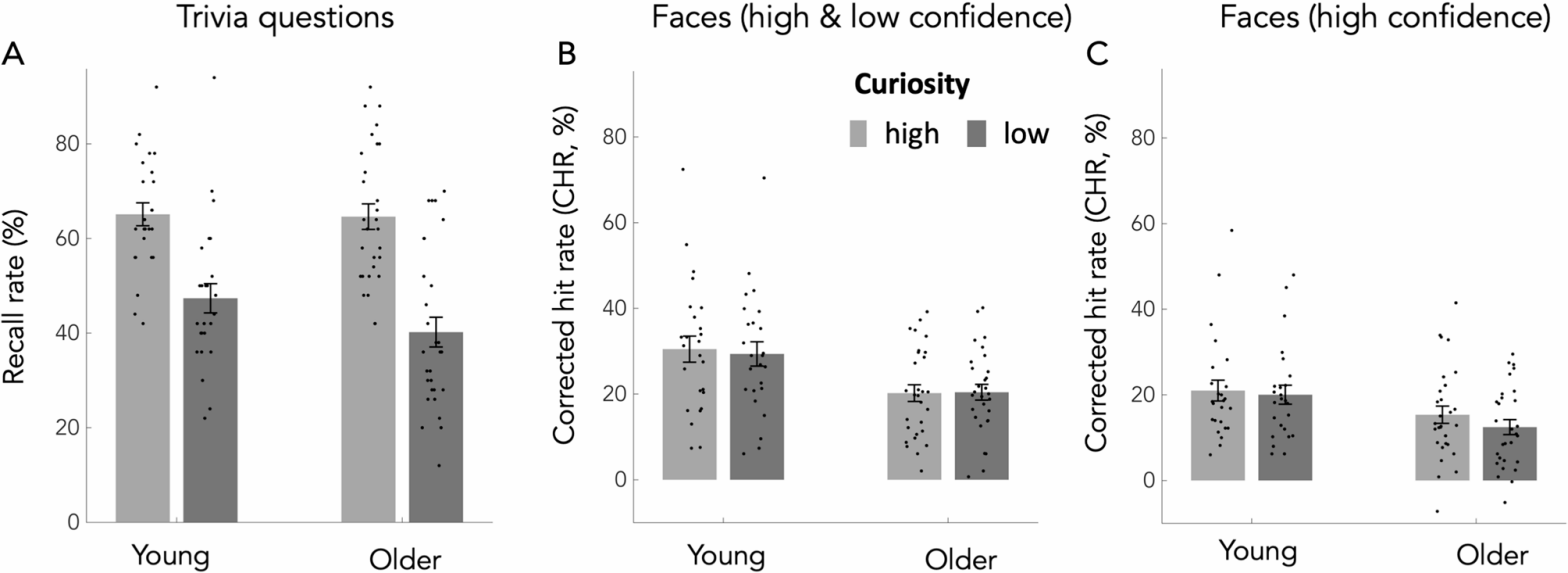
Experiment 1, results for the effect of state EC on memory performance separately for trivia questions (**A**) and faces (**B**, **C**). High state EC was associated with better recall for trivia questions (main effect of curiosity) with only anecdotal evidence in favor of group differences (i.e. interaction of curiosity × group, see text). High state EC did not increase recognition memory for faces that were presented in between questions and answers (**B**, **C**). Accordingly, there was no main effect of state EC on corrected hit rates (CHR) when both high and low confidence response were considered together (B) or when focusing on high confidence responses alone (**C**). In both cases (**B**, **C**), recognition memory was better in young adults (main effect of age but no interaction with curiosity; see text for further explanation). Error bars correspond to one standard error of the mean.

A 2 × 2 ANOVA on free recall rates with the factors age (young, older) and state EC (high, low) revealed no main effect of age (F(1,52) = 1.05, *p* = 0.31, η^2^_p_ = 0.02), but a significant main effect of state EC (F(1,52) = 165.18, *p* < 0.001, η^2^_p_ = 0.76), and a marginally significant interaction of age × state EC (F(1,52) = 4.11, *p* = 0.048, η^2^_p_ = 0.07). The main effect of state EC was driven by higher recall rates in the high state EC condition; the interaction was driven by a marginally more pronounced difference between high and low curiosity recall rates in older participants, as revealed by post hoc t-test comparing both differences (t(52)=-2.03; *p* = 0.048). An additional 2 × 2 Bayes ANOVA with the factors age (young, older) and state EC (high, low) confirmed and completed the picture (Table S2). It revealed a BF_10_ = 6.92e+14 for the main effect of curiosity (indicating extreme evidence for an effect of curiosity), a BF_10_ = 0.36 for the main effect of age (indicating anecdotal to moderate evidence against an effect of age), and a BF_10_ = 1.41 for the interaction (indicating anecdotal evidence in favour of it)^58,59^. See Fig. 2A.

#### Recognition memory for faces

In a first step, recognition memory was analyzed using a 2 × 2 ANOVA with the factor age (young, older) and state EC (high, low) on the sum of both high and low confidence responses (i.e. CHR). It showed a significant main effect of age (F(1,52) = 9.45, *p* = 0.003, η^2^_p_ = 0.15), which was driven by higher CHR in young adults, but no main effect of state EC (F(1,52) = 0.113, *p* > 0.7, η^2^_p_ = 0.002), and no interaction (F(1,52) = 0.238, *p* > 0.6, η^2^_p_ = 0.005). An additional 2 × 2 Bayes ANOVA (Table S3) revealed a BF_10_ = 11.44 for the main effect of age (indicating strong evidence for an effect), a BF_10_ = 0.21 for the main effect of curiosity (moderate evidence against an effect), and a BF_10_ = 0.29 for the interaction of curiosity × age (moderate evidence against an interaction effect).

In a second step, we only used high confidence CHR employing a 2 × 2 ANOVA. It also revealed a main effect of age (F(1,52) = 6.04, *p* = 0.017, η^2^_p_ = 0.104), but no significant main effect of state EC (F(1,52) = 2.36, *p* > 0.1, η^2^_p_ = 0.04) and no significant interaction (F(1,52) = 0.6, *p* > 0.44, η^2^_p_ = 0.01). An additional 2 × 2 Bayes ANOVA (Table S4) revealed a BF_10_ = 3.48 for the main effect of age (moderate evidence for an effect), a BF_10_ = 0.62 for the main effect of curiosity (anecdotal evidence against an effect), and a BF_10_ = 0.35 for the interaction of curiosity × age (anecdotal to moderate evidence against the interaction effect).

Taken together, while state EC enhanced free recall similarly in both age groups, there was no convincing evidence for an effect of state EC on recognition memory for faces that were presented between the curiosity inducing questions and answers. See Fig. 2B-C.

#### Relationship of trait curiosity and memory advantage

**Fig. 3:**
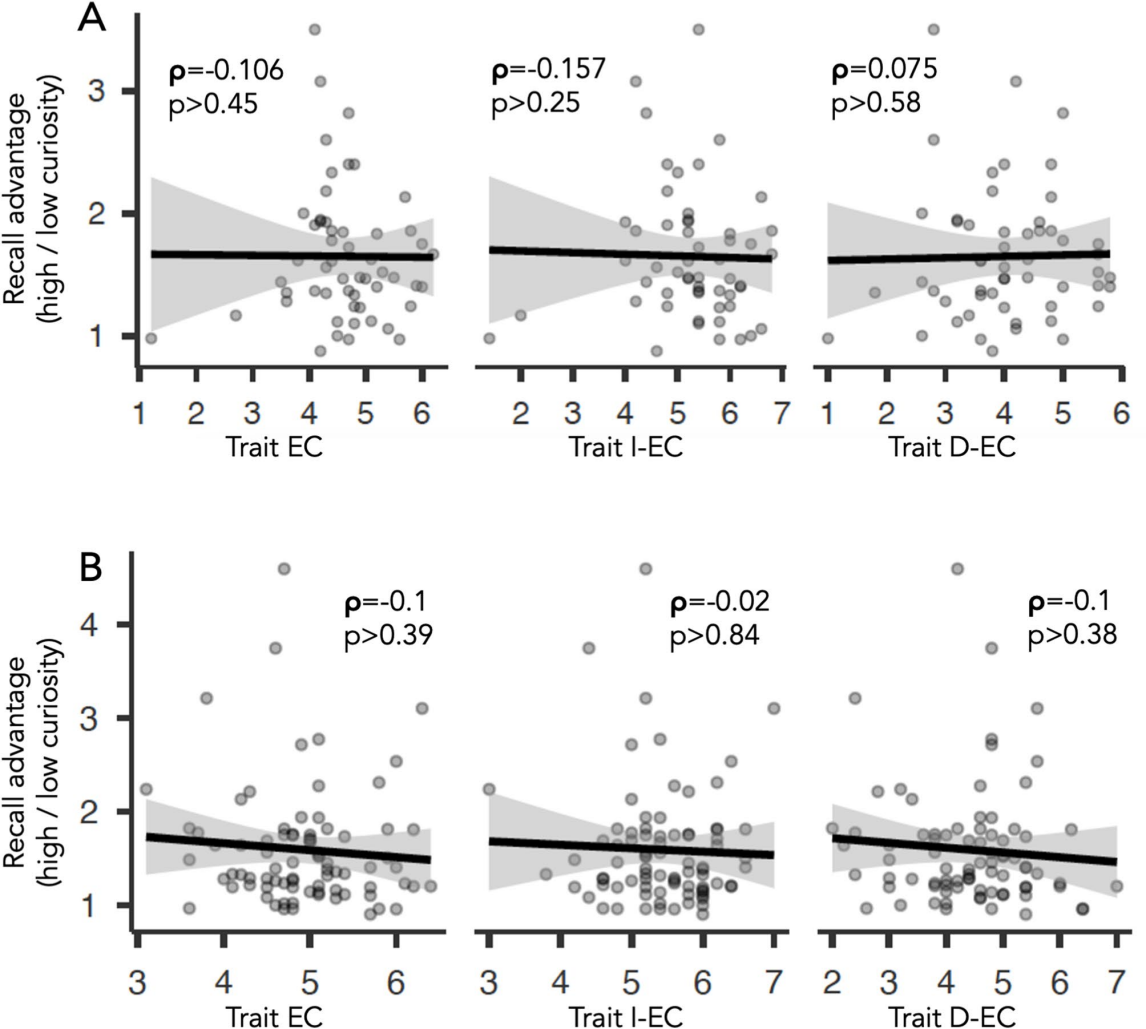
Results of a correlation between trait EC and memory advantage by state EC on trivia questions. The ratio of recall rates for high / low state EC did not correlate with any type of trait curiosity across both age groups in either experiment 1 (**A**) or experiment 2 (**B**). I-EC abbreviates interest type of trait epistemic curiosity, D-EC abbreviates deprivation type of trait epistemic curiosity. Shown are the correlation coefficient rho (ρ) and p-values (p).

In a next step, we tested whether the memory advantage by state EC on trivia questions (i.e. ratio of recall rates for high / low curiosity) also related to trait curiosity. Here, we used the average values for trait EC, I-EC and D-EC, respectively. None of the three correlations revealed a significant effect (Fig. 3A, *p* > 0.25). An additional Bayesian correlation complemented these observations by showing moderate evidence against a correlation for (average) trait EC (τ = -0.07, BF_10_ = 0.24), and D-EC (τ = 0.05, BF_10_ = 0.2), but only anecdotal evidence for I-EC (τ = -0.12, BF_10_ = 0.37). Note that we averaged values across both age groups since the initial 2 × 2 ANOVA did not reveal a robust group by curiosity interaction and to increase the power of our analysis. Mean values of both groups (young, older) did not differ in I-EC (young = 5.41, SD = 1.07, older = 5.13, SD = 0.96, *p* = 0.32, BF_10_ = 0.42), but overall trait EC (young = 4.93, SD = 0.98, older = 4.37, SD = 0.64, *p* = 0.015, BF_10_ = 4.43) and D-EC (young = 4.45, SD = 1.07, older = 3.69, SD = 0.87, *p* < 0.006, BF_10_ = 7.31).

### Experiment 2

#### Recall performance on trivia questions

**Fig. 4:**
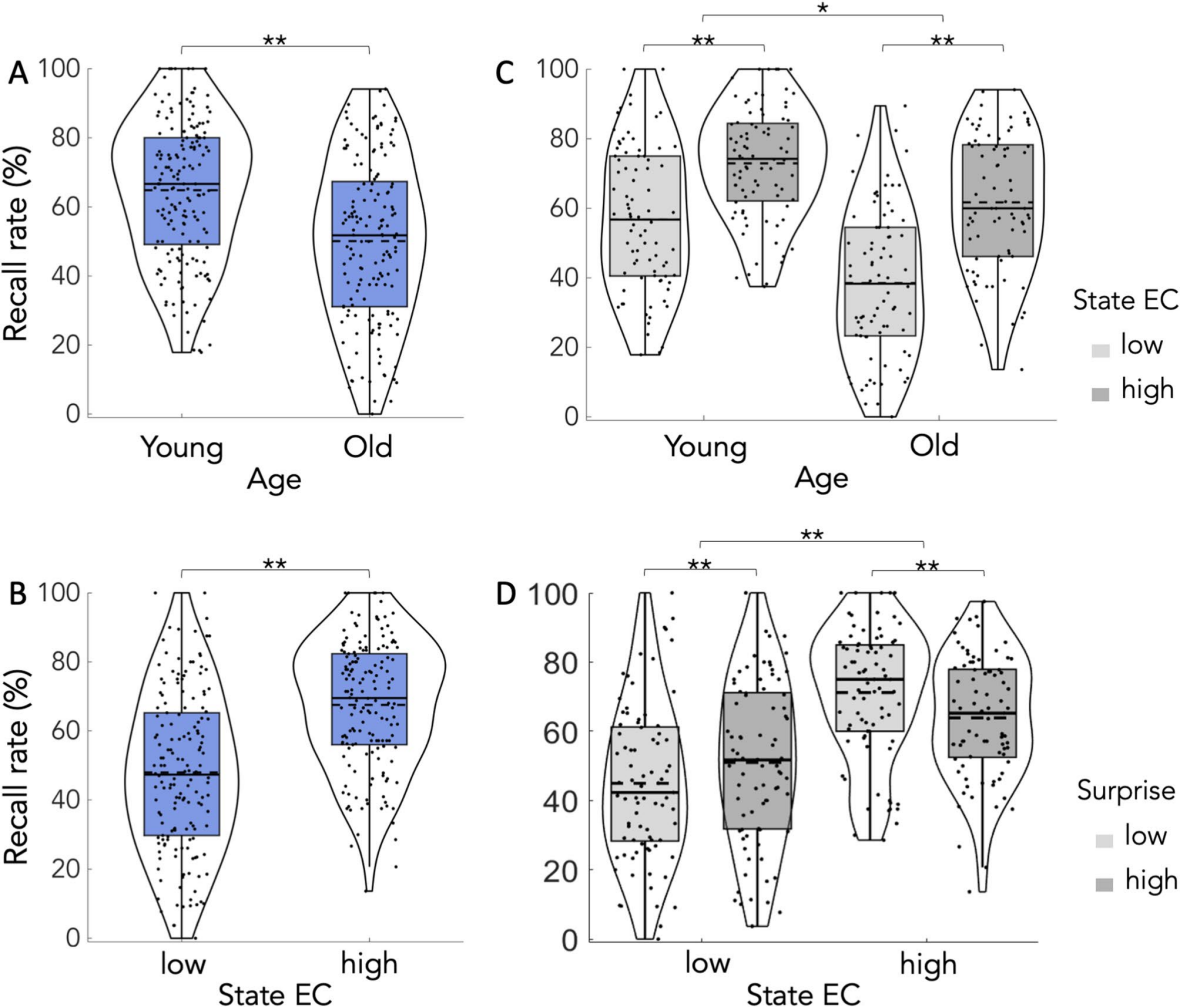
Experiment 2, results for the effects of age, state EC and surprise on memory performance for trivia questions. We observed a main effect of age (**A**), a main effect of state EC (**B**), an interaction of age × state EC (**C**), and an interaction of surprise × curiosity (**D**). **indicates *p* < 0.001, *indicates *p* < 0.01. See text for more information.

A 2 × 2 × 2 ANOVA on free recall rates with the factors age (young, older), state EC (high, low) and surprise (high, low) revealed a main effect of age (F(1,79) = 21.80, *p* < 0.001, η^2^_p_ = 0.22), which was driven by higher recall rates in young adults (*p* < 0.001), a main effect of curiosity (F(1,79) = 215.1, *p* < 0.001, η^2^_p_ = 0.73), which was driven by higher recall rates in the high curiosity condition (*p* < 0.001), but no significant main effect of surprise (F(1,79) = 0.12, *p* = 0.74, η^2^_p_ = 0.001). The additional 2 × 2 × 2 Bayes ANOVA revealed a BF_10_ = 1369.61 for the main effect of age (extreme evidence for an effect), a BF_10_ = 7.66e+20 for the main effect of state EC (extreme evidence for an effect), and a BF_10_ = 0.13 for the main effect of surprise (moderate evidence against an effect). See Fig. 4A, B and Table S5.

**Fig. 5:**
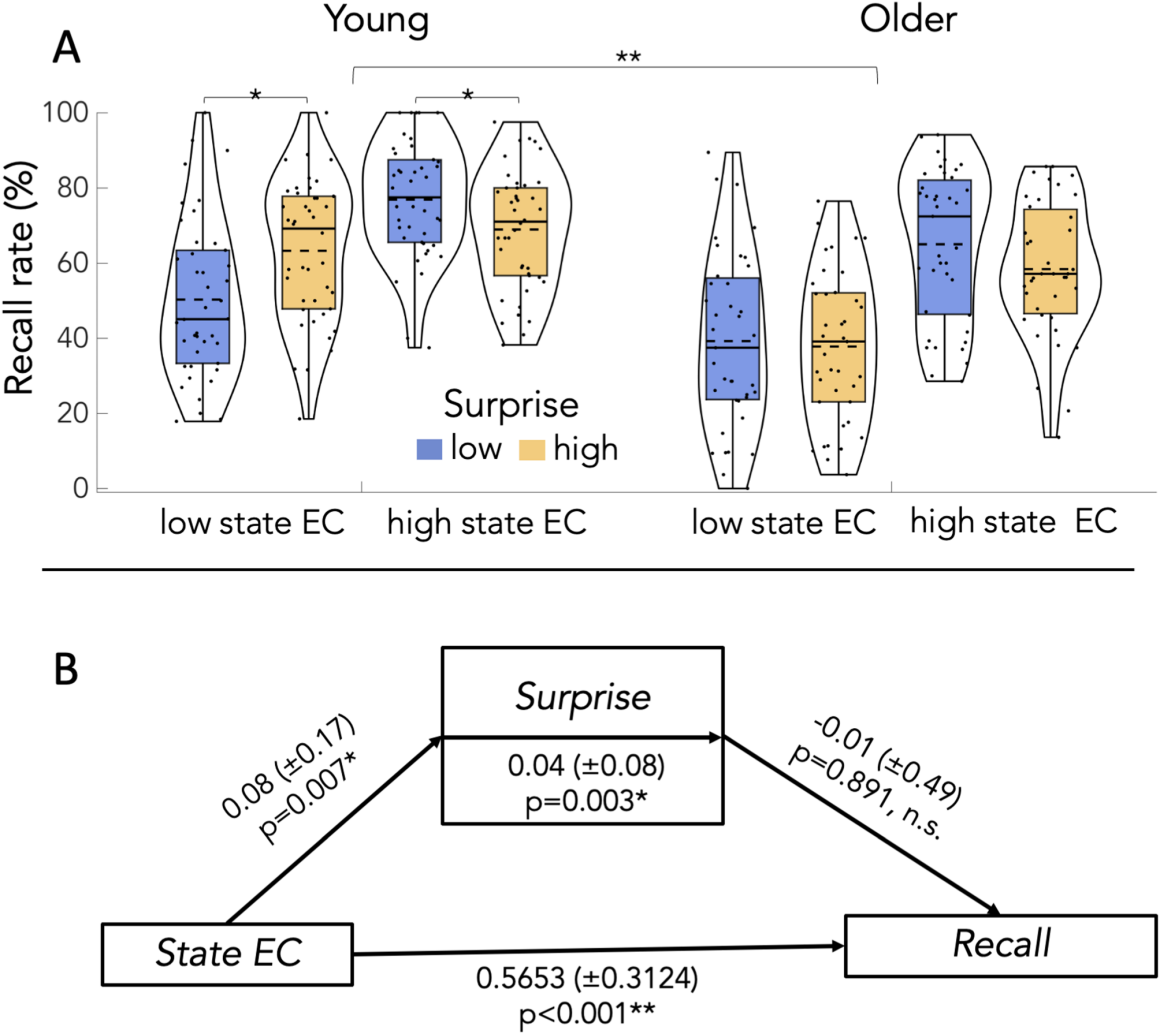
Experiment 2, results for the effects of age, state EC and surprise on memory performance for trivia questions. **A**, there was a significant three-way interaction of age, state EC and surprise, which was driven by a positive effect of surprise in the low curiosity condition in young but not older adults, and a negative effect of surprise in the high curiosity condition in young but not older adults. **B**, a logistic mediation analysis in young adults revealed that state EC had a significant direct effect on recall performance and surprise. Additionally, state EC had an indirect effect on recall performance via surprise indicating surprise-mediated benefits for the ability to recall answers to curious questions. Shown are mean mediation coefficients and standard deviations (in brackets). **indicates *p* < 0.001, *indicates *p* < 0.01, n.s. abbreviates “not significant”.

Interactions: the same frequentist 2 × 2 × 2 ANOVA also revealed a significant interaction of state EC × age (F(1,79) = 6.95, *p* = 0.01, η^2^_p_ = 0.08), which was driven by a more pronounced curiosity effects on recall rates in older adults (Fig. 4C, mean difference in young = 16.13 vs. mean difference in older adults = 23.20), a significant interaction of surprise × state EC (F(1,79) = 32.89, *p* < 0.001, η^2^_p_ = 0.29), which was driven by an opposing effect of surprise depending on the degree of state EC (Fig. 4D), and – importantly – a significant three-way interaction of state EC × surprise × age (F(1,79) = 12.01, *p* < 0.001, η^2^_p_ = 0.13, Fig. 5). The interaction of surprise × group was not significant (*p* > 0.14).

To further disentangle the effects behind the three-way interaction (Fig. 5), two follow up 2 × 2 ANOVAs with the factors state EC (high, low) and surprise (high, low) were performed for young and older subjects, separately. For the young adults, it revealed a main effect of state EC (F(1,41) = 95.39; *p* < 0.001; η^2^_p_ = 0.7), which was driven by higher recall rates in the high state EC condition (*p* < 0.001), no main effect of surprise (F(1,41) = 0.59; *p* = 0.45; η^2^_p_ = 0.01), but a significant interaction of state EC and surprise (F(1,41) = 41.75; *p* < 0.001; η^2^_p_ = 0.5). Post hoc analyses revealed a positive effect of high surprise on recall in the low state EC condition (*p* = 0.003), but a negative effect of high surprise on recall in the high state EC condition (*p* = 0.012, see Fig. 5). Both post hoc comparisons remained significant after Bonferroni-Holm correction, *p* < 0.05. A 2 × 2 Bayes ANOVA completed the picture (Table S6). It revealed a BF_10_ = 8.10e+6 for the main effect of state EC (extreme evidence for an effect), a BF_10_ = 0.25 for a main effect of surprise (moderate evidence against an effect), and a BF_10_ = 1279.35 for the interaction of curiosity × surprise (indicating extreme evidence for an interaction effect).

In older adults, the same 2 × 2 ANOVA revealed a significant main effect of state EC (F(1,38) = 117.45; *p* < 0.001; η^2^_p_ = 0.76), driven by higher recall rates in the high curiosity condition (*p* < 0.001), but no main effect of surprise (F(1,38) = 1.77; *p* = 0.19; η^2^_p_ = 0.04), and no significant interaction of state EC and surprise (F(1,38) = 2.63; *p* = 0.11; η^2^_p_ = 0.065). A 2 × 2 Bayes ANOVA for older adults partly confirmed and completed the picture (Table S7). It revealed a BF_10_ = 1.02e+14 for the main effect of state EC (extreme evidence for an effect), a BF_10_ = 0.38 for a main effect of surprise (anecdotal to moderate evidence against an effect), and a BF_10_ = 0.39 for the interaction of curiosity × surprise (indicating anecdotal evidence against an interaction effect).

Taken together, state EC enhanced free recall in both age groups despite lower overall memory in older adults. The three-way interaction and post hoc analyses indicate a differential effect of surprise and state EC in the young, which is reduced in older adults. See Fig. 5A.

#### Recognition memory for faces

In a first step, recognition memory was analyzed using a 2 × 2 × 2 ANOVA with the factors age (young, older), state EC (high, low), and surprise (high, low) on the sum of both high and low confidence responses (CHR). To ensure a meaningful interpretation of the data, only those subjects with a CHR > 0 (in each of the four conditions) were included, which reduces the sample to *n* = 36 young and *n* = 33 older adults. It revealed a mean effect of age (F(1,67) = 14.1; *p* < 0.001; η^2^_p_ = 0.11), driven by better recognition memory in young adults (*p* < 0.001), but no other main effects or interactions (all p’s > 0.19). This was confirmed by a 2 × 2 × 2 Bayes measure ANOVA. Briefly, it revealed a BF_10_ = 75.6 for the main effect of age (very strong evidence for an effect), a BF_10_ = 0.24 for the main effect of state EC (moderate evidence against an effect), and a BF_10_ = 0.13 for the main effect of surprise (moderate evidence against an effect). All other statistics can be found in Table S8.

We repeated the 2 × 2 × 2 ANOVA on high-confidence CHR and, again, included only those subjects with a CHR > 0 (in each of the four condition), which reduced that sample to *n* = 36 young and *n* = 28 older adults. The analysis did not reveal any significant main effects or interactions (all p’s > 0.2). Again, this was confirmed and complemented by a 2 × 2 × 2 Bayes ANOVA. Briefly, it revealed a BF_10_ = 0.4 for the main effect of age (anecdotal evidence against an effect), a BF_10_ = 0.13 for the main effect of curiosity (moderate evidence against an effect), and a BF_10_ = 0.13 for the main effect of surprise (also moderate evidence against an effect). All other statistics can be found in Table S9.

Taken together, our analyses provide evidence against an effect of state EC or surprise on recognition memory for faces that were presented between the curiosity inducing questions and corresponding answers.

#### Relationship of trait curiosity and memory advantage

In a next step, a possible relationship of the memory advantage by state EC on trivia questions and trait curiosity was tested. Following experiment 1, we used the ratio of recall rates for high / low state EC but this time averaged across high and low surprise (reflecting the main effect of state EC); these individual values were correlated with values for overall trait EC, I-EC and D-EC, respectively. Similar to experiment 1, none of the three correlations revealed a significant effect (all p’s > 0.38, Fig. 3B). Again, this was confirmed by a Bayesian correlation with moderate evidence against an effect for (average) trait EC (τ =-0.08, BF_10_ = 0.24), I-EC (τ =-0.01, BF_10_ = 0.15), and D-EC (τ =-0.07, BF_10_ = 0.21). Note that mean values of both groups (young, older) did not differ in overall trait EC (young = 5, SD = 0.7, old = 4.86, SD = 0.7, *p* = 0.38, BF_10_ = 0.32), I-EC (young = 5.41, SD = 0.68, old = 5.47, SD = 0.71, *p* = 0.69, BF_10_ = 0.25), or D-EC (young = 4.59, SD = 0.98, old = 4.25, SD = 1.08, *p* = 0.15, BF_10_ = 0.58).

#### Mediation analysis

Based on the significant interaction of state EC and surprise in young (but not older adults, Fig. 5A), we investigated whether surprise mediated the relationship of state EC on LTM for answers to curious questions (i.e. recall). To this end, we performed a logistic mediation analysis with state EC as predictor, surprise as mediator and recall rate as dependent variable (see methods). The resulting mediation coefficients were then analyzed at the group level using one-sample t-tests. It revealed a significant direct effect of state EC on recall performance (t(39) = 11.45, *p* < 0.001, d = 1.81), a significant direct effect of state EC on surprise (t(39) = 2.85, *p* = 0.007, d = 0.45), and, importantly, a significant indirect effect of state EC on recall through surprise (t(39) = 3.18, *p* = 0.003, d = 0.503). The direct effect of surprise on recall was not significant (t(39)=-0.138, *p* = 0.891, d=-0.022). See Fig. 5B.

### Experiment 3

#### Correlation analysis

**Fig. 6:**
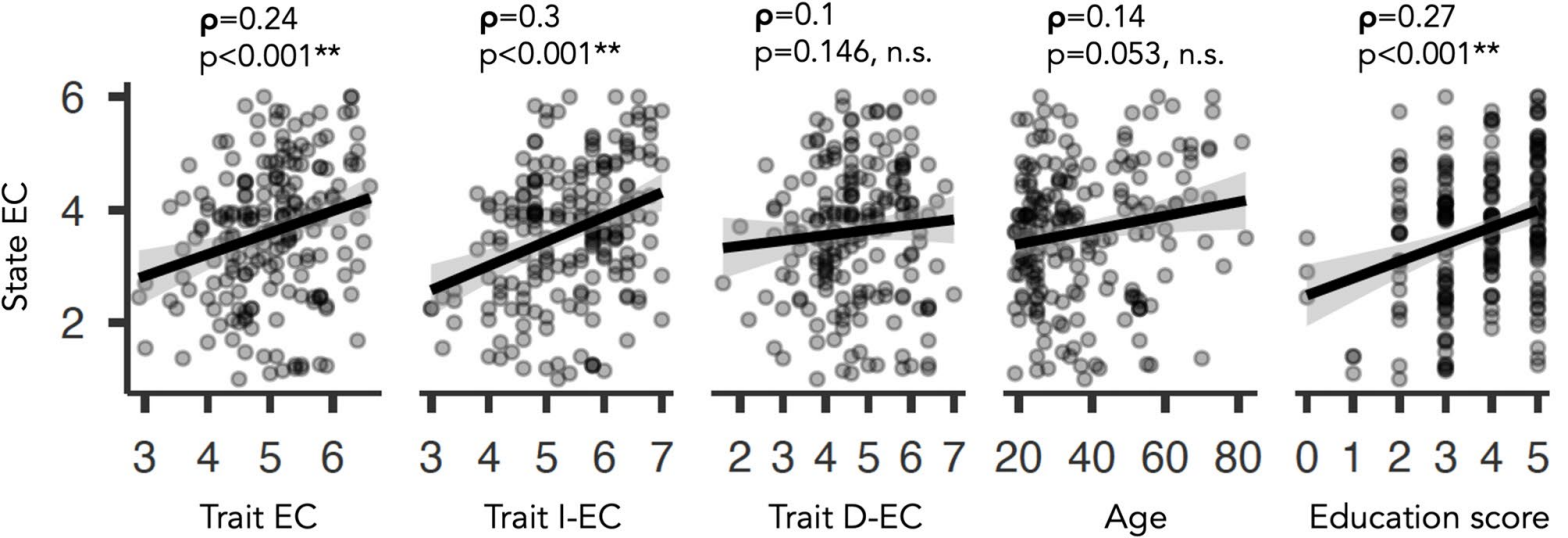
Experiment 3, results of a correlation analysis. State EC correlated with overall trait EC, trait I-EC, and education score, but not with trait D-EC and age. Shown are correlation coefficients (rho, ρ) and p-values (p). **indicates *p* < 0.001, n.s. abbreviates “not significant”.

In a first step, a correlation analysis was performed with all variables of interest, including (average) trait EC, trait I-EC, trait D-EC, state EC, “education score” (see methods), and age (see Table S10). Importantly, when focusing on the separate dimensions of trait EC, a significant correlation was only observed for I-EC and state EC (*p* < 0.001, ρ = 0.3) but not D-EC and state EC (*p* = 0.15, ρ = 0.1), see Fig. 6. There was no significant correlation between age and state EC (*p* > 0.05, ρ = 0.14) or age and any type of trait curiosity (all p’s > 0.05). The correlation between I-EC and state curiosity remained significant with age as covariate in a partial correlation (*p* < 0.001). For “education score”, there was a highly significant correlation with state EC (*p* < 0.001, ρ = 0.27), and a marginally significant correlation with trait I-EC (*p* = 0.01, ρ = 0.18) and age (*p* = 0.04, ρ = 0.15) but no significant relationship with trait D-EC (*p* > 0.36, ρ=-0.07).

An additional Bayesian correlation (see Table S10) confirmed a robust relationship between state EC and (average) trait EC (τ = 0.17, BF_10_ = 35.9), state EC and I-EC (τ = 0.21, BF_10_ = 1312.6), state EC and “education score” (τ = 0.21, BF_10_ = 801). Most of the other effects were only moderate or inconclusive, including trait I-EC and “education score” (τ = 0.14, BF_10_ = 7.89), state EC and trait D-EC (τ = 0.08, BF_10_ = 0.32), age and (average) trait EC (τ = 0.025, BF_10_ = 0.1), age and “education score” (τ = 0.11, BF_10_ = 1.32), age and state EC (τ = 0.09, BF_10_ = 0.6), age and trait I-EC (τ =-0.075, BF_10_ = 0.31), as well as age and trait D-EC (τ = 0.09, BF_10_ = 0.53).

#### Relationship of state and trait curiosity with education score

**Fig. 7:**
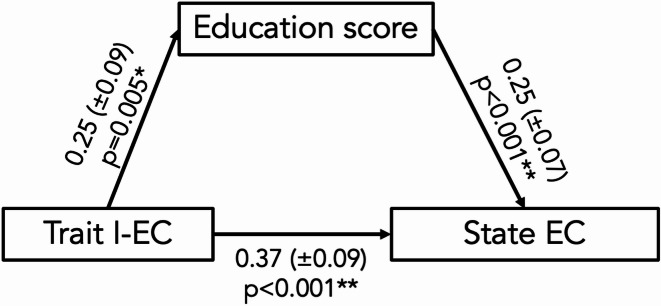
Experiment 3, results of a mediation analysis with state EC as dependent variable, trait I-EC as predictor and education score as mediator. State EC could be predicted directly via trait I-EC and indirectly via education score. Shown are path estimates and significance values. **indicates *p* < 0.001, *indicates *p* < 0.01.

To further test the relationship between state EC and trait EC with formal education, we performed a mediation analysis with trait curiosity (I-EC) as predictor, “education score” as mediator and state EC as dependent variable. The overall model was significant (total effect, *p* < 0.001, see Table S11); importantly, both the direct and indirect effect were also significant (*p* < 0.05, see Fig. 7 for path estimates). Note that bootstrapping with 1000 samples largely confirmed this observation (Table S12).

## Discussion

State epistemic curiosity (EC) can promote long-term memory in healthy young and older adults. This is surprising given that the underlying neurobiological structures, including the dopaminergic mesolimbic system, typically degenerate with age. One possibility is that other psychological factors, especially surprise, play a role. Moreover, the triadic interplay of state EC, trait EC and formal education across the adult lifespan remains unclear. Here, we addressed these open questions in two laboratory studies (experiment 1 and 2) and an online survey (experiment 3). Our data confirm that state EC drives LTM for relevant information in young and healthy older adults (experiment 1 and 2), and, importantly, this effect was further modulated by surprise, depending on age (experiment 2). More specifically, in young adults surprise enhanced free recall in a state of low EC but it reduced it in a state of high EC (interaction), which is compatible with a quadratic effect of arousal on memory performance. In older adults, only curiosity had a significant effect on free recall. This suggests that state EC can enhance free recall across the adult lifespan, but the differential effects of surprise decrease with age (experiment 2). Finally, trait I-EC had a direct and an indirect effect via formal education on state EC (experiment 3). Taken together, these findings give novel insights into how state and trait EC, together with surprise, differentially contribute to LTM and relate to educational attainment.

In experiment 1, we could replicate previous work^24,25^ by showing that being in a state of high EC promotes subsequent LTM in young and older adults (Fig. 2A). From a conceptual point of view, this positive effect has been hypothesized to relate to the intrinsic drive to close an “information gap”, that is associated with uncertainty, and therefore new knowledge acquisition^6^. In other words, closing an “information gap” has an intrinsic value since it reduces uncertainty about the unknown through knowledge acquisition. From a physiological point of view, state curiosity effects on memory most likely relate to enhanced activity in the dopaminergic mesolimbic system, including the SN/VTA, nucleus accumbens and hippocampus^12–14,34^. In fact, dopamine is a potent neuromodulator that, through a release from midbrain structures to the MTL, promotes synaptic plasticity and therefore memory encoding^63–65^. Importantly, the structural integrity of the mesolimbic system and interrelated brain regions typically declines with age^19,20,66^, which can lead to changes in how motivational information is being processed^23^ with direct consequences for LTM performance^22^. For instance, healthy older adults exhibited an abnormal reward prediction error signal in the nucleus accumbens, which, together with task performance, can be restored by dopaminergic stimulation^67^. Taken together, despite our successful replication^24,25 ^a positive effect of state EC is at odds with imaging studies demonstrating structural and functional age-related changes of the dopaminergic mesolimbic system and a typical memory decline with age.

Experiment 2 helps to integrate these apparently contradictory views by suggesting that surprise, in addition to state EC, serves as a key component. Indeed, outstanding items, distinct features or unique characteristics are known to enhance memory via changes in neural activity in memory related networks, including the mesolimbic system^68^. For instance, rare words written in capital letters are subsequently remembered better as compared to much more frequent words written in small font – an effect known as the “von Restorff effect” or isolation effect^69–71^. From a more computational point of view, surprise is characterized by a deviation from expectation and experience, which closely relates to the concepts of predictive coding^16,17^. Therefore, novelty processing (which constitutes a first essential step in memory encoding), surprise and prediction errors, that appear to change with age, are closely related at a behavioral, neural and computational level^27–30^. Our findings extend these views by suggesting that the effects of state EC and surprise are not additive but follow an inverted U-shape, at least in young adults (Fig. 5A, left panel). This is in line with notions of cognitive performance going hand in hand with an optimal (or intermediate) level of arousal (here, high curiosity and low surprise); if arousal is low (here, low curiosity and low surprise) or too high (here, high curiosity and high surprise), then memory performance is rather low (Fig. 5A, left panel). Indeed, inverted U-shaped relationships, often associated with the Yerkes-Dodson Law^72 ^describe how varying levels of arousal can influence cognitive performance, including LTM^73^. Neurobiological models^74^ are in line with such a view and further suggest that neurotransmitters, especially norepinephrine^75 ^could play a significant role. The reduction of an inverted U-shape relationship in older adults (Fig. 5A, right panel) could be explained by changes in attentional processing due to age-related degeneration of the locus coeruleus, a brain region that releases norepinephrine and dopamine into the hippocampus to also modulate memory processes^66,76^.

In older adults, there was only an effect of curiosity on memory but no convincing evidence for an interaction with surprise (Fig. 5A, right panel). This further underlines conceptual and possibly neurobiological differences of curiosity and surprise^28,30 ^and it indicates that only state EC can enhance free recall across the adult lifespan, but the effects of surprise appear to be reduced with age. This, in turn, can be explained on the basis of two conceptions. First, semantic knowledge typically increases with age, which results in more accurate generative models of the world. Therefore, deviations from predictions can lead to smaller prediction errors in healthy older adults as compared to their younger counterparts^31^. At the same time, smaller prediction errors go hand-in-hand with affective changes (i.e. less negative affect and possibly lower arousal levels), which also helps to explain why the effect of curiosity is less modulated by surprise in older adults. Second, due to impaired cognitive abilities, older adults may have problems in properly detecting deviations from predictions^31^. This, however, is less likely since (high vs. low) surprise levels could be quantified not only by young but also older adults. An important consideration for our interpretation here relates to the underlying statistics. While the frequentist and Bayes 2 × 2 × 2 ANOVAs clearly show evidence in favor of a three-way interaction, the separate 2 × 2 Bayes ANOVA in older adults does not provide compelling evidence against the interaction of state EC and surprise. Therefore, state EC enhanced free recall in both age groups despite lower memory performance in older adults. However, the differential effects of surprise and state EC in the young are only reduced but not completely absent in the older (Fig. 5).

The results of experiment 2 (Fig. 5B) further suggest that surprise mediates the relationship between state EC and memory, despite not directly predicting recall. This implies that surprise enhances memory specifically in the context of heightened curiosity, potentially by amplifying attention or salience during learning. From a methodological point of view, this indirect-only effect is consistent with contemporary mediation models^77,78^, which recognize that a significant indirect pathway can exist even when the mediator does not independently predict the outcome. These findings also align with a previous fMRI study in young adults, showing that a mediating effect of surprise relies on the rostrolateral prefrontal cortex^29^, which is integral to uncertainty driven exploration^79,80^. Future studies, however, need to address whether age-related structural or functional brain changes underlie the observed group differences in our study. These would require not only a comparison between different age groups, but they should also use state-of-the art imaging techniques, possibly in combination with psychopharmacological means. Specifically, a combination of functional and structural markers of age-related brain changes could be used to investigate their relationship with behavior^81–83 ^and pharmacological means would allow to test the role of different neuromodulators in this context^84^.

As expected, there was (across all subjects) a significant relationship of state and trait EC, that was primarily driven by I-EC but not D-EC (Fig. 6, experiment 3). This finding provides further empirical evidence for a conceptual distinction of interest vs. deprivation type trait EC^35 ^and it is consistent with previous research on trait and state showing that people with higher self-reported trait levels also exhibit higher levels of corresponding trait-relevant states more often in daily life^85,86^. This, in turn, can have an impact on cognitive abilities as suggested by the notion that „intellectual curiosity“, i.e. typical intellectual engagement, is, together with intelligence and conscientiousness, a third predictor of academic performance^36^. Along these lines, our mediation analysis (Fig. 7, experiment 3) revealed that trait I-EC exerts both a direct and an indirect effect (via educational achievement) on state EC. Therefore, highly curious individuals may experience situational curiosity more often (or more intensely) partially because trait curiosity promotes higher educational attainment, which in turn enhances state curiosity. This finding is compatible with previous work suggesting a dynamic interplay between curiosity, cognitive engagement and learning-related outcomes^41,42^. Thus, educational achievement may represent a developmental pathway linking stable curiosity traits to everyday curiosity states. Note, however, that all three variables (state EC, trait I-EC and educational degree) were assessed simultaneously, precluding clear inferences about temporal order or causality. Future research should, therefore, use longitudinal designs to better capture the temporal dynamics and clarify causal relationships (see below for further discussions).

With regard to age-related differences in state and trait EC, our findings are less conclusive (experiment 3). Only for average trait EC, the frequentist and Bayes approach revealed moderate to strong evidence for the absence of a significant correlation between age and trait EC, which is in line with the notion of trait being (by definition) a rather stable property. For age and state EC, age and trait I-EC, as well as age and trait D-EC, the evidence in favor of or against a correlation was inconclusive (i.e. *p* > 0.05 and BF_10_ between 0.31 and 0.6). While several studies suggested age-related curiosity changes^2,46^, it is also clear that different concepts or types of curiosity imply a more complex picture^4,87–90^. For instance, older adults with lower levels in trait curiosity as compared to their younger counterparts exhibited higher levels in state curiosity when interacting with robots depending on personal relevance^49^. Therefore, age-dependent changes in state and trait EC need to be further investigated possibly taking into account concepts from cognitive neuroscience indicating that compensatory mechanisms help to explain distinct developmental trajectories^84^.

There was no effect of state EC on subsequent memory for faces that were presented between a curiosity inducing question and subsequently presented answer (Fig. 1, experiment 1 and 2). Although this is at odds with some previous studies using similar designs, it fits to the observation that a positive effect of state curiosity on memory is much more pronounced for relevant (i.e. answers) as compared to incidental (i.e. faces) information^12,25^. It has been argued that close temporal proximity is a key factor^55^, which could not be replicated here. In fact, in experiment 1 the inter-stimulus interval (ISI) between a question and face picture was 4000 ms; in experiment 2, it was limited to 2000 ms, which, in a recent publication^55 ^showed the strongest effect. Although the Bayesian evidence against an effect of curiosity on faces was moderate in our experiment 1 and strong in experiment 2, future studies should further investigate which specific aspects of state EC promote memory for incidental information^34^.

Finally, we would like to point out the following strengths and limitations. First, our experiments are based on cross-sectional designs. While this offers a time and cost-efficient comparison between age-groups, individual changes and developmental trajectories can only be inferred. However, cross-sectional (compared to longitudinal) approaches typically suffer less from practice effects and drop-outs (i.e. attrition), which could potentially lead to a selection bias. Second, our online study was economical, flexible and reached out to a larger population than our laboratory studies. However, we had no control over subjects’ physical environment, including technical issues, lighting conditions, or other distractors, which may have induced noise in our data. Third, a stronger effect of state EC on LTM in older adults (experiment 2) was unexpected; this finding could be due to motivational differences between groups or other cognitive aspects, such as learning strategies. In any case, it is difficult to be explained post hoc and should, therefore, be replicated in an independent sample. Fourth, our rationale and theoretical frameworks include the assumption of structural and possibly functional brain changes. This, however, can only be investigated using brain imaging techniques, such as MRI, EEG or PET. These aspects, together with larger samples and more balanced age-distributions (especially in experiment 3) should be considered in future work.

To conclude, state curiosity promotes long-term memory for relevant information throughout the adult lifespan. Importantly, this effect was modulated by surprise in young but less in older adults, and, finally, formal education mediated the relationship between trait I-EC and state EC. As such, our findings offer new understandings of how state and trait curiosity, together with surprise, facilitate learning and knowledge acquisition across the adult lifespan.

## Supplementary Information

Below is the link to the electronic supplementary material.

Supplementary Material 1

## Data Availability

All data are available via OSF (https://osf.io/y2mjv/).
