# Supplementary material for: Curiosity and surprise differentially affect memory depending on age
Source: Sci Rep. 2025 Sep 12;15:32423. doi: 10.1038/s41598-025-14479-x (PMC12432193; doi:10.1038/s41598-025-14479-x)
Supplement: Supplementary file 1 — Supplementary Material 1 [file 41598_2025_14479_MOESM1_ESM.docx]

**Supplementary material**

**Curiosity and surprise differentially affect memory**

**depending on age**

Alexandra Sobczak^1, *^, Tineke Steiger^1, *^, Marthe Mieling^1^, Nico Bunzeck^1,2 *^

^1^ Department of Psychology, University of Lübeck, Germany

^2^ Center of Brain, Behavior and Metabolism, University of Lübeck, Germany

^a^ corresponding author, [nico.bunzeck@uni-luebeck.de](mailto:nico.bunzeck@uni-luebeck.de)

^*^These authors contributed equally to this work.

**Table S1**. Recall performance for answers to trivia questions in experiment 1 (A) and experiment 2 (B). Shown are absolute numbers for each condition (means and standard deviations in brackets) for each group separately.

| **A** – Experiment 1 | **high curiosity** | **low curiosity** |
| --- | --- | --- |
| **Young** | 32.6 (6.07) | 23.7 (7.73) |
| **Old** | 32.3 (7.26) | 20.1 (8.47) |

| **B** – Experiment 2 | **high curiosity / high surprise** | **high curiosity / low surprise** | **low curiosity / high surprise** | **low curiosity / low surprise** |
| --- | --- | --- | --- | --- |
| **Young** | 16.38 (6.47) | 20.24 (7.04) | 12.43 (5.09) | 14.10 (6.05) |
| **Old** | 13.67 (4.98) | 17.67 (8.25) | 9.18 (5.33) | 9.28 (6.39) |

**Table S2.** Experiment 1, results of a 2x2 Bayesian ANOVA with the factors age (young, older) and state EC (high, low) on recall rate. Note that a model only including the interaction term Curiosity * Age is not reported separately in jamovi and, therefore, needs to be calculated by comparing the BF_10_ of the model with the interaction against the BF_10_ of the model with only the two main effects ^1^. Here: BF_10_ Curiosity ✻ Age = 5.36e+14 / 3.80e+14 = 1.41.

| Model Comparison | | | | | | | | | | | |
| --- | --- | --- | --- | --- | --- | --- | --- | --- | --- | --- | --- |
| **Models** | | **P(M)** | | **P(M\|data)** | | **BF_M_** | | **BF_10_** | | **error %** | |
| Null model (incl. subject) |  | 0.200 |  | 6.22e-16 |  | 2.49e-15 |  | 1.000 |  |  |  |
| Curiosity |  | 0.200 |  | 0.430 |  | 3.02 |  | 6.92e+14 |  | 0.980 |  |
| Age |  | 0.200 |  | 2.24e-16 |  | 8.96e-16 |  | 0.360 |  | 0.898 |  |
| Curiosity + Age |  | 0.200 |  | 0.236 |  | 1.24 |  | 3.80e+14 |  | 1.222 |  |
| Curiosity + Age + Curiosity ✻ Age |  | 0.200 |  | 0.333 |  | 2.00 |  | 5.36e+14 |  | 1.748 |  |
| Note. All models include subject. | | | | | | | | | | | |
|  | | | | | | | | | | | |

| Analysis of Effects | | | | | | | |
| --- | --- | --- | --- | --- | --- | --- | --- |
| **Effects** | | **P(incl)** | | **P(incl\|data)** | | **BF_Inclusion_** | |
| Curiosity |  | 0.600 |  | 1.000 |  | 8.58e+14 |  |
| Age |  | 0.600 |  | 0.570 |  | 0.883 |  |
| Curiosity ✻ Age |  | 0.200 |  | 0.333 |  | 2.001 |  |
|  | | | | | | | |

**Table S3.** Experiment 1, results of a 2x2 Bayesian ANOVA with the factors age (young, older) and state EC (high, low) on the sum of both high and low confidence responses (CRH) for faces. Note that a model only including the interaction term Curiosity * Age is not reported separately in jamovi and, therefore, needs to be calculated by comparing the BF_10_ of the model with the interaction against the BF_10_ of the model with only the two main effects ^1^. Here: BF_10_ Curiosity ✻ Age = 0.694 / 2.408 = 0.288.

| Model Comparison | | | | | | | | | | | |
| --- | --- | --- | --- | --- | --- | --- | --- | --- | --- | --- | --- |
| **Models** | | **P(M)** | | **P(M\|data)** | | **BF_M_** | | **BF_10_** | | **error %** | |
| Null model (incl. subject) |  | 0.200 |  | 0.0635 |  | 0.2712 |  | 1.000 |  |  |  |
| curiosity |  | 0.200 |  | 0.0131 |  | 0.0532 |  | 0.207 |  | 1.02 |  |
| age |  | 0.200 |  | 0.7265 |  | 10.6237 |  | 11.443 |  | 2.82 |  |
| curiosity + age |  | 0.200 |  | 0.1528 |  | 0.7217 |  | 2.408 |  | 2.79 |  |
| curiosity + age + curiosity ✻ age |  | 0.200 |  | 0.0441 |  | 0.1844 |  | 0.694 |  | 2.35 |  |
| Note. All models include subject. | | | | | | | | | | | |
|  | | | | | | | | | | | |

| Analysis of Effects | | | | | | | |
| --- | --- | --- | --- | --- | --- | --- | --- |
| **Effects** | | **P(incl)** | | **P(incl\|data)** | | **BF_Inclusion_** | |
| curiosity |  | 0.600 |  | 0.2100 |  | 0.177 |  |
| age |  | 0.600 |  | 0.9234 |  | 8.035 |  |
| curiosity ✻ age |  | 0.200 |  | 0.0441 |  | 0.184 |  |
|  | | | | | | | |

**Table S4.** Experiment 1, results of a 2x2 Bayesian ANOVA with the factors age (young, older) and state EC (high, low) on high-confidence CHR for faces. Note that a model only including the interaction term Curiosity * Age is not reported separately in jamovi and, therefore, needs to be calculated by comparing the BF_10_ of the model with the interaction against the BF_10_ of the model with only the two main effects ^1^. Here: BF_10_ Curiosity ✻ Age = 0.718 / 2.037 = 0.352.

| Model Comparison | | | | | | | | | | | |
| --- | --- | --- | --- | --- | --- | --- | --- | --- | --- | --- | --- |
| **Models** | | **P(M)** | | **P(M\|data)** | | **BF_M_** | | **BF_10_** | | **error %** | |
| Null model (incl. subject) |  | 0.200 |  | 0.1274 |  | 0.584 |  | 1.000 |  |  |  |
| curiosity |  | 0.200 |  | 0.0785 |  | 0.341 |  | 0.616 |  | 1.67 |  |
| age |  | 0.200 |  | 0.4431 |  | 3.183 |  | 3.477 |  | 5.35 |  |
| curiosity + age |  | 0.200 |  | 0.2596 |  | 1.402 |  | 2.037 |  | 1.68 |  |
| curiosity + age + curiosity ✻ age |  | 0.200 |  | 0.0914 |  | 0.403 |  | 0.718 |  | 3.37 |  |
| Note. All models include subject. | | | | | | | | | | | |
|  | | | | | | | | | | | |

| Analysis of Effects | | | | | | | |
| --- | --- | --- | --- | --- | --- | --- | --- |
| **Effects** | | **P(incl)** | | **P(incl\|data)** | | **BF_Inclusion_** | |
| curiosity |  | 0.600 |  | 0.4295 |  | 0.502 |  |
| age |  | 0.600 |  | 0.7941 |  | 2.571 |  |
| curiosity ✻ age |  | 0.200 |  | 0.0914 |  | 0.403 |  |
|  | | | | | | | |

**Table S5.** Experiment 2, results of a 2x2x2 Bayesian ANOVA with the factors age (young, older), state EC (high, low), and surprise (high, low) on recall rate.

| Model Comparison | | | | | | | | | | | |
| --- | --- | --- | --- | --- | --- | --- | --- | --- | --- | --- | --- |
| **Models** | | **P(M)** | | **P(M\|data)** | | **BF_M_** | | **BF_10_** | | **error %** | |
| Null model (incl. subject) |  | 0.0526 |  | 3.44e-27 |  | 6.20e-26 |  | 1.000 |  |  |  |
| Curiosity |  | 0.0526 |  | 2.64e0-6 |  | 4.75e0-5 |  | 7.66e+20 |  | 1.41 |  |
| Surprise |  | 0.0526 |  | 4.45e-28 |  | 8.00e-27 |  | 0.129 |  | 3.19 |  |
| Curiosity + Surprise |  | 0.0526 |  | 3.33e0-7 |  | 5.99e0-6 |  | 9.67e+19 |  | 2.95 |  |
| Curiosity + Surprise + Curiosity ✻ Surprise |  | 0.0526 |  | 7.33e0-5 |  | 0.00132 |  | 2.13e+22 |  | 1.96 |  |
| Age |  | 0.0526 |  | 4.72e-24 |  | 8.49e-23 |  | 1369.611 |  | 11.80 |  |
| Curiosity + Age |  | 0.0526 |  | 0.00380 |  | 0.06864 |  | 1.10e+24 |  | 1.47 |  |
| Surprise + Age |  | 0.0526 |  | 5.29e-25 |  | 9.52e-24 |  | 153.631 |  | 3.46 |  |
| Curiosity + Surprise + Age |  | 0.0526 |  | 4.98e0-4 |  | 0.00897 |  | 1.45e+23 |  | 3.53 |  |
| Curiosity + Surprise + Curiosity ✻ Surprise + Age |  | 0.0526 |  | 0.10788 |  | 2.17667 |  | 3.13e+25 |  | 3.21 |  |
| Curiosity + Age + Curiosity ✻ Age |  | 0.0526 |  | 0.00445 |  | 0.08055 |  | 1.29e+24 |  | 2.48 |  |
| Curiosity + Surprise + Age + Curiosity ✻ Age |  | 0.0526 |  | 6.01e0-4 |  | 0.01083 |  | 1.75e+23 |  | 3.94 |  |
| Curiosity + Surprise + Curiosity ✻ Surprise + Age + Curiosity ✻ Age |  | 0.0526 |  | 0.15401 |  | 3.27685 |  | 4.47e+25 |  | 8.88 |  |
| Surprise + Age + Surprise ✻ Age |  | 0.0526 |  | 2.68e-25 |  | 4.83e-24 |  | 77.944 |  | 2.82 |  |
| Curiosity + Surprise + Age + Surprise ✻ Age |  | 0.0526 |  | 4.22e0-4 |  | 0.00759 |  | 1.22e+23 |  | 3.01 |  |
| Curiosity + Surprise + Curiosity ✻ Surprise + Age + Surprise ✻ Age |  | 0.0526 |  | 0.10596 |  | 2.13335 |  | 3.08e+25 |  | 3.90 |  |
| Curiosity + Surprise + Age + Curiosity ✻ Age + Surprise ✻ Age |  | 0.0526 |  | 5.87e0-4 |  | 0.01057 |  | 1.70e+23 |  | 15.23 |  |
| Curiosity + Surprise + Curiosity ✻ Surprise + Age + Curiosity ✻ Age + Surprise ✻ Age |  | 0.0526 |  | 0.15910 |  | 3.40563 |  | 4.62e+25 |  | 5.01 |  |
| Curiosity + Surprise + Curiosity ✻ Surprise + Age + Curiosity ✻ Age + Surprise ✻ Age + Curiosity ✻ Surprise ✻ Age |  | 0.0526 |  | 0.46261 |  | 15.49525 |  | 1.34e+26 |  | 12.30 |  |
| Note. All models include subject. | | | | | | | | | | | |
|  | | | | | | | | | | | |

| Analysis of Effects | | | | | | | |
| --- | --- | --- | --- | --- | --- | --- | --- |
| **Effects** | | **P(incl)** | | **P(incl\|data)** | | **BF_Inclusion_** | |
| Curiosity |  | 0.7368 |  | 1.000 |  | 1.61e+15 |  |
| Surprise |  | 0.7368 |  | 0.992 |  | 42.90 |  |
| Age |  | 0.7368 |  | 1.000 |  | 4680.59 |  |
| Curiosity ✻ Surprise |  | 0.3158 |  | 0.990 |  | 206.87 |  |
| Curiosity ✻ Age |  | 0.3158 |  | 0.781 |  | 7.74 |  |
| Surprise ✻ Age |  | 0.3158 |  | 0.729 |  | 5.82 |  |
| Curiosity ✻ Surprise ✻ Age |  | 0.0526 |  | 0.463 |  | 15.50 |  |
|  | | | | | | | |

**Table S6.** Experiment 2, results of a 2x2 Bayesian ANOVA for young subjects with the factors state EC (high, low), and surprise (high, low) on free recall rates. Note that a model only including the interaction term Curiosity * Age is not reported separately in jamovi and, therefore, needs to be calculated by comparing the BF_10_ of the model with the interaction against the BF_10_ of the model with only the two main effects ^1^. Here: BF_10_ Curiosity ✻ Age = 3.16e+9 / 2.47e+6 = 1279.35.

| Model Comparison | | | | | | | | | | | |
| --- | --- | --- | --- | --- | --- | --- | --- | --- | --- | --- | --- |
| **Models** | | **P(M)** | | **P(M\|data)** | | **BF_M_** | | **BF_10_** | | **error %** | |
| Null model (incl. subject) |  | 0.200 |  | 3.15e-10 |  | 1.26e-9 |  | 1.000 |  |  |  |
| curiosity |  | 0.200 |  | 0.00255 |  | 0.01023 |  | 8.10e+6 |  | 1.72 |  |
| surprise |  | 0.200 |  | 7.86e-11 |  | 3.15e-10 |  | 0.250 |  | 3.02 |  |
| curiosity + surprise |  | 0.200 |  | 7.79e0-4 |  | 0.00312 |  | 2.47e+6 |  | 10.02 |  |
| curiosity + surprise + curiosity ✻ surprise |  | 0.200 |  | 0.99667 |  | 1196.91400 |  | 3.16e+9 |  | 1.86 |  |
| Note. All models include subject. | | | | | | | | | | | |
|  | | | | | | | | | | | |

| Analysis of Effects | | | | | | | |
| --- | --- | --- | --- | --- | --- | --- | --- |
| **Effects** | | **P(incl)** | | **P(incl\|data)** | | **BF_Inclusion_** | |
| curiosity |  | 0.600 |  | 1.000 |  | 1.69e+9 |  |
| surprise |  | 0.600 |  | 0.997 |  | 261 |  |
| curiosity ✻ surprise |  | 0.200 |  | 0.997 |  | 1197 |  |
|  | | | | | | | |

**Table S7**. Experiment 2, results of a 2x2 Bayesian ANOVA for older subjects with the factors state EC (high, low), and surprise (high, low) on free recall rates. Note that a model only including the interaction term Curiosity * Age is not reported separately in jamovi and, therefore, needs to be calculated by comparing the BF_10_ of the model with the interaction against the BF_10_ of the model with only the two main effects ^1^. Here: BF_10_ Curiosity ✻ Age = 2.59e+13 / 6.57e+13 = 0.394.

| Model Comparison | | | | | | | | | | | |
| --- | --- | --- | --- | --- | --- | --- | --- | --- | --- | --- | --- |
| **Models** | | **P(M)** | | **P(M\|data)** | | **BF_M_** | | **BF_10_** | | **error %** | |
| Null model (incl. subject) |  | 0.200 |  | 5.17e-15 |  | 2.07e-14 |  | 1.000 |  |  |  |
| Curiosity |  | 0.200 |  | 0.526 |  | 4.447 |  | 1.02e+14 |  | 1.01 |  |
| Surprise |  | 0.200 |  | 1.94e-15 |  | 7.77e-15 |  | 0.376 |  | 4.52 |  |
| Curiosity + Surprise |  | 0.200 |  | 0.339 |  | 2.055 |  | 6.57e+13 |  | 1.51 |  |
| Curiosity + Surprise + Curiosity ✻ Surprise |  | 0.200 |  | 0.134 |  | 0.620 |  | 2.59e+13 |  | 2.14 |  |
| Note. All models include subject. | | | | | | | | | | | |
|  | | | | | | | | | | | |

| Analysis of Effects | | | | | | | |
| --- | --- | --- | --- | --- | --- | --- | --- |
| **Effects** | | **P(incl)** | | **P(incl\|data)** | | **BF_Inclusion_** | |
| Curiosity |  | 0.600 |  | 1.000 |  | 9.69e+13 |  |
| Surprise |  | 0.600 |  | 0.474 |  | 0.600 |  |
| Curiosity ✻ Surprise |  | 0.200 |  | 0.134 |  | 0.620 |  |
|  | | | | | | | |

**Table S8.** Experiment 2, results of a 2x2x2 Bayesian ANOVA with the factors age (young, older), state EC (high, low), and surprise (high, low) on the sum of both high and low confidence responses (CRH) for faces.

| Model Comparison | | | | | | | | | | | |
| --- | --- | --- | --- | --- | --- | --- | --- | --- | --- | --- | --- |
| **Models** | | **P(M)** | | **P(M\|data)** | | **BF_M_** | | **BF_10_** | | **error %** | |
| Null model (incl. subject) |  | 0.0526 |  | 0.00863 |  | 0.15671 |  | 1.00000 |  |  |  |
| Curiosity |  | 0.0526 |  | 0.00207 |  | 0.03740 |  | 0.24026 |  | 1.986 |  |
| Surprise |  | 0.0526 |  | 0.00115 |  | 0.02079 |  | 0.13366 |  | 0.799 |  |
| Curiosity + Surprise |  | 0.0526 |  | 2.77e-4 |  | 0.00499 |  | 0.03212 |  | 2.098 |  |
| Curiosity + Surprise + Curiosity ✻ Surprise |  | 0.0526 |  | 5.67e-5 |  | 0.00102 |  | 0.00657 |  | 4.421 |  |
| Age |  | 0.0526 |  | 0.65245 |  | 33.79071 |  | 75.59230 |  | 2.955 |  |
| Curiosity + Age |  | 0.0526 |  | 0.15975 |  | 3.42222 |  | 18.50867 |  | 5.357 |  |
| Surprise + Age |  | 0.0526 |  | 0.09242 |  | 1.83307 |  | 10.70830 |  | 2.720 |  |
| Curiosity + Surprise + Age |  | 0.0526 |  | 0.02132 |  | 0.39210 |  | 2.47000 |  | 4.614 |  |
| Curiosity + Surprise + Curiosity ✻ Surprise + Age |  | 0.0526 |  | 0.00428 |  | 0.07746 |  | 0.49642 |  | 8.473 |  |
| Curiosity + Age + Curiosity ✻ Age |  | 0.0526 |  | 0.03048 |  | 0.56583 |  | 3.53103 |  | 7.710 |  |
| Curiosity + Surprise + Age + Curiosity ✻ Age |  | 0.0526 |  | 0.00374 |  | 0.06766 |  | 0.43384 |  | 5.927 |  |
| Curiosity + Surprise + Curiosity ✻ Surprise + Age + Curiosity ✻ Age |  | 0.0526 |  | 7.00e-4 |  | 0.01260 |  | 0.08107 |  | 4.555 |  |
| Surprise + Age + Surprise ✻ Age |  | 0.0526 |  | 0.01700 |  | 0.31137 |  | 1.97008 |  | 3.092 |  |
| Curiosity + Surprise + Age + Surprise ✻ Age |  | 0.0526 |  | 0.00388 |  | 0.07007 |  | 0.44928 |  | 4.448 |  |
| Curiosity + Surprise + Curiosity ✻ Surprise + Age + Surprise ✻ Age |  | 0.0526 |  | 8.81e-4 |  | 0.01588 |  | 0.10212 |  | 16.173 |  |
| Curiosity + Surprise + Age + Curiosity ✻ Age + Surprise ✻ Age |  | 0.0526 |  | 6.98e-4 |  | 0.01258 |  | 0.08092 |  | 3.853 |  |
| Curiosity + Surprise + Curiosity ✻ Surprise + Age + Curiosity ✻ Age + Surprise ✻ Age |  | 0.0526 |  | 1.27e-4 |  | 0.00229 |  | 0.01472 |  | 4.217 |  |
| Curiosity + Surprise + Curiosity ✻ Surprise + Age + Curiosity ✻ Age + Surprise ✻ Age + Curiosity ✻ Surprise ✻ Age |  | 0.0526 |  | 7.12e-5 |  | 0.00128 |  | 0.00825 |  | 6.140 |  |
| Note. All models include subject. | | | | | | | | | | | |
|  | | | | | | | | | | | |

| Analysis of Effects | | | | | | | |
| --- | --- | --- | --- | --- | --- | --- | --- |
| **Effects** | | **P(incl)** | | **P(incl\|data)** | | **BF_Inclusion_** | |
| Curiosity |  | 0.7368 |  | 0.22834 |  | 0.10568 |  |
| Surprise |  | 0.7368 |  | 0.14662 |  | 0.06136 |  |
| Age |  | 0.7368 |  | 0.98781 |  | 28.93496 |  |
| Curiosity ✻ Surprise |  | 0.3158 |  | 0.00612 |  | 0.01334 |  |
| Curiosity ✻ Age |  | 0.3158 |  | 0.03582 |  | 0.08049 |  |
| Surprise ✻ Age |  | 0.3158 |  | 0.02266 |  | 0.05023 |  |
| Curiosity ✻ Surprise ✻ Age |  | 0.0526 |  | 7.12e-5 |  | 0.00128 |  |
|  | | | | | | | |

**Table S9.** Experiment 2, results of a 2x2x2 Bayesian ANOVA with the factors age (young, older), state EC (high, low), and surprise (high, low) on high confidence responses (high-confidence CRH) for faces.

| Model Comparison | | | | | | | | | | | |
| --- | --- | --- | --- | --- | --- | --- | --- | --- | --- | --- | --- |
| **Models** | | **P(M)** | | **P(M\|data)** | | **BF_M_** | | **BF_10_** | | **error %** | |
| Null model (incl. subject) |  | 0.0526 |  | 0.54142 |  | 21.25152 |  | 1.00000 |  |  |  |
| surprise |  | 0.0526 |  | 0.07299 |  | 1.41736 |  | 0.13482 |  | 1.214 |  |
| curiosity |  | 0.0526 |  | 0.07297 |  | 1.41686 |  | 0.13478 |  | 0.974 |  |
| surprise + curiosity |  | 0.0526 |  | 0.01014 |  | 0.18439 |  | 0.01873 |  | 4.680 |  |
| surprise + curiosity + surprise ✻ curiosity |  | 0.0526 |  | 0.00384 |  | 0.06931 |  | 0.00708 |  | 3.910 |  |
| Age |  | 0.0526 |  | 0.21698 |  | 4.98782 |  | 0.40076 |  | 0.824 |  |
| surprise + Age |  | 0.0526 |  | 0.02814 |  | 0.52121 |  | 0.05198 |  | 1.018 |  |
| curiosity + Age |  | 0.0526 |  | 0.02963 |  | 0.54966 |  | 0.05473 |  | 2.520 |  |
| surprise + curiosity + Age |  | 0.0526 |  | 0.00400 |  | 0.07224 |  | 0.00738 |  | 3.389 |  |
| surprise + curiosity + surprise ✻ curiosity + Age |  | 0.0526 |  | 0.00156 |  | 0.02805 |  | 0.00287 |  | 3.511 |  |
| surprise + Age + surprise ✻ Age |  | 0.0526 |  | 0.00880 |  | 0.15972 |  | 0.01624 |  | 1.866 |  |
| surprise + curiosity + Age + surprise ✻ Age |  | 0.0526 |  | 0.00119 |  | 0.02146 |  | 0.00220 |  | 2.445 |  |
| surprise + curiosity + surprise ✻ curiosity + Age + surprise ✻ Age |  | 0.0526 |  | 4.66e-4 |  | 0.00839 |  | 8.61e-4 |  | 2.592 |  |
| curiosity + Age + curiosity ✻ Age |  | 0.0526 |  | 0.00627 |  | 0.11355 |  | 0.01158 |  | 2.557 |  |
| surprise + curiosity + Age + curiosity ✻ Age |  | 0.0526 |  | 8.82e-4 |  | 0.01589 |  | 0.00163 |  | 5.604 |  |
| surprise + curiosity + surprise ✻ curiosity + Age + curiosity ✻ Age |  | 0.0526 |  | 3.38e-4 |  | 0.00609 |  | 6.25e-4 |  | 4.855 |  |
| surprise + curiosity + Age + surprise ✻ Age + curiosity ✻ Age |  | 0.0526 |  | 2.70e-4 |  | 0.00487 |  | 4.99e-4 |  | 6.360 |  |
| surprise + curiosity + surprise ✻ curiosity + Age + surprise ✻ Age + curiosity ✻ Age |  | 0.0526 |  | 9.93e-5 |  | 0.00179 |  | 1.83e-4 |  | 4.522 |  |
| surprise + curiosity + surprise ✻ curiosity + Age + surprise ✻ Age + curiosity ✻ Age + surprise ✻ curiosity ✻ Age |  | 0.0526 |  | 2.70e-5 |  | 4.86e-4 |  | 4.99e-5 |  | 8.785 |  |
| Note. All models include subject. | | | | | | | | | | | |
|  | | | | | | | | | | | |

| Analysis of Effects | | | | | | | |
| --- | --- | --- | --- | --- | --- | --- | --- |
| **Effects** | | **P(incl)** | | **P(incl\|data)** | | **BF_Inclusion_** | |
| surprise |  | 0.7368 |  | 0.13273 |  | 0.0547 |  |
| curiosity |  | 0.7368 |  | 0.13167 |  | 0.0542 |  |
| Age |  | 0.7368 |  | 0.29864 |  | 0.1521 |  |
| surprise ✻ curiosity |  | 0.3158 |  | 0.00632 |  | 0.0138 |  |
| surprise ✻ Age |  | 0.3158 |  | 0.01085 |  | 0.0238 |  |
| curiosity ✻ Age |  | 0.3158 |  | 0.00789 |  | 0.0172 |  |
| surprise ✻ curiosity ✻ Age |  | 0.0526 |  | 2.70e-5 |  | 4.86e-4 |  |
|  | | | | | | | |

**Table S10.** Results experiment 3, correlation analysis.

| Correlation Matrix | | | | | | | | | | | | | | | |
| --- | --- | --- | --- | --- | --- | --- | --- | --- | --- | --- | --- | --- | --- | --- | --- |
|  | |  | | **Trait EC** | | **Trait I-EC** | | **Trait D-EC** | | **Age** | | **State EC** | | **Education score** | |
| Trait EC |  | Spearman's rho |  | — |  |  |  |  |  |  |  |  |  |  |  |
|  |  | p-value |  | — |  |  |  |  |  |  |  |  |  |  |  |
| Trait I-EC |  | Spearman's rho |  | 0.764 | *** | — |  |  |  |  |  |  |  |  |  |
|  |  | p-value |  | < .001 |  | — |  |  |  |  |  |  |  |  |  |
| Trait D-EC |  | Spearman's rho |  | 0.817 | *** | 0.288 | *** | — |  |  |  |  |  |  |  |
|  |  | p-value |  | < .001 |  | < .001 |  | — |  |  |  |  |  |  |  |
| Age |  | Spearman's rho |  | 0.039 |  | -0.107 |  | 0.129 |  | — |  |  |  |  |  |
|  |  | p-value |  | 0.584 |  | 0.134 |  | 0.071 |  | — |  |  |  |  |  |
| State EC |  | Spearman's rho |  | 0.240 | *** | 0.301 | *** | 0.104 |  | 0.139 |  | — |  |  |  |
|  |  | p-value |  | < .001 |  | < .001 |  | 0.146 |  | 0.053 |  | — |  |  |  |
| Education score |  | Spearman's rho |  | 0.053 |  | 0.180 | * | -0.067 |  | 0.146 | * | 0.270 | *** | — |  |
|  |  | p-value |  | 0.459 |  | 0.012 |  | 0.351 |  | 0.041 |  | < .001 |  | — |  |
| Note. * p < .05, ** p < .01, *** p < .001 | | | | | | | | | | | | | | | |
|  | | | | | | | | | | | | | | | |

| Bayesian Kendall's Tau | | | | | | | | | | | | | | | | | | | | |  |
| --- | --- | --- | --- | --- | --- | --- | --- | --- | --- | --- | --- | --- | --- | --- | --- | --- | --- | --- | --- | --- | --- |
|  | | **BF₁₀** | **Trait EC** | | | **Trait I-EC** | | | **Trait D-EC** | | | **Age** | | | **State EC** | | | **Education score** | | |  |
| Trait EC |  | Kendall's tau |  | — |  | |  |  | |  |  | |  |  | |  |  | |  |  | |
|  | | BF₁₀ |  | — |  | |  |  | |  |  | |  |  | |  |  | |  |  | |
| Trait I-EC |  | Kendall's tau |  | 0.5992 | *** | | — |  | |  |  | |  |  | |  |  | |  |  | |
|  | | BF₁₀ |  | 3.29e+32 |  | | — |  | |  |  | |  |  | |  |  | |  |  | |
| Trait D-EC |  | Kendall's tau |  | 0.6581 | *** | | 0.2130 | *** | | — |  | |  |  | |  |  | |  |  | |
|  | | BF₁₀ |  | 2.70e+39 |  | | 1632.269 |  | | — |  | |  |  | |  |  | |  |  | |
| Age |  | Kendall's tau |  | 0.0250 |  | | -0.0748 |  | | 0.0898 |  | | — |  | |  |  | |  |  | |
|  | | BF₁₀ |  | 0.107 |  | | 0.312 |  | | 0.532 |  | | — |  | |  |  | |  |  | |
| State EC |  | Kendall's tau |  | 0.1662 | ** | | 0.2106 | *** | | 0.0755 |  | | 0.0929 |  | | — |  | |  |  | |
|  | | BF₁₀ |  | 35.890 |  | | 1312.603 |  | | 0.319 |  | | 0.601 |  | | — |  | |  |  | |
| Education score |  | Kendall's tau |  | 0.0422 |  | | 0.1439 |  | | -0.0522 |  | | 0.1110 |  | | 0.206 | *** | | — |  | |
|  | | BF₁₀ |  | 0.137 |  | | 7.896 |  | | 0.168 |  | | 1.315 |  | | 801 |  | | — |  | |
| Note. * BF₁₀ > 10, ** BF₁₀ > 30, *** BF₁₀ > 100 | | | | | | | | | | | | | | | | | | | | |  |

**Table S11.** Results experiment 3, mediation analysis. Listed are the estimates for the mediation analysis on state EC as dependent variable, trait curiosity (I-EC) as predictor and “education score” as mediator.

| Mediation Estimates | | | | | | | | | | | | | | | | | |  |  |  |  |  |  |
| --- | --- | --- | --- | --- | --- | --- | --- | --- | --- | --- | --- | --- | --- | --- | --- | --- | --- | --- | --- | --- | --- | --- | --- |
| **Effect** | | **Estimate** | | | | | | **SE** | | **Z** | | | | **p** | | | |  |  |  |  |  |  |
| Indirect |  | 0.0615 | | | |  | | 0.0280 |  | 2.20 | |  | | 0.028 | |  | |  |  |  |  |  |  |
| Direct |  | 0.3744 | | | |  | | 0.0884 |  | 4.24 | |  | | < .001 | |  | |  |  |  |  |  |  |
| Total |  | 0.4360 | | | |  | | 0.0894 |  | 4.88 | |  | | < .001 | |  | |  |  |  |  |  |  |
|  | | | | | | | | | | | | | | | | | |  |  |  |  |  |  |
| Path Estimates | | | | | | | | | | | | | | | | | | | | | | | |
|  | | | |  | | |  | | | | | | **Estimate** | | | | **SE** | | | **Z** | | **p** | |
| Trait (I-EC) | | |  | → |  | | Education score | | | |  | | 0.252 | |  | | 0.0902 | |  | 2.79 |  | 0.005 |  |
| Education score | | |  | → |  | | State EC | | | |  | | 0.245 | |  | | 0.0688 | |  | 3.55 |  | < .001 |  |
| Trait (I-EC) | | |  | → |  | | State EC | | | |  | | 0.374 | |  | | 0.0884 | |  | 4.24 |  | < .001 |  |
|  | | | | | | | | | | | | | | | | | | | | | | | |

**Table S12.** Results experiment 3, mediation analysis; as in table S11 but here with bootstrapping (1000 samples).

| Mediation Estimates | | | | | | | | | | | | | | | | | |  |  |  |  |  |  |
| --- | --- | --- | --- | --- | --- | --- | --- | --- | --- | --- | --- | --- | --- | --- | --- | --- | --- | --- | --- | --- | --- | --- | --- |
| **Effect** | | **Estimate** | | | | | | **SE** | | **Z** | | | | **p** | | | |  |  |  |  |  |  |
| Indirect |  | 0.0615 | | | |  | | 0.0319 |  | 1.93 | |  | | 0.054 | |  | |  |  |  |  |  |  |
| Direct |  | 0.3744 | | | |  | | 0.0872 |  | 4.29 | |  | | < .001 | |  | |  |  |  |  |  |  |
| Total |  | 0.4360 | | | |  | | 0.0855 |  | 5.10 | |  | | < .001 | |  | |  |  |  |  |  |  |
|  | | | | | | | | | | | | | | | | | |  |  |  |  |  |  |
| Path Estimates | | | | | | | | | | | | | | | | | | | | | | | |
|  | | | |  | | |  | | | | | | **Estimate** | | | | **SE** | | | **Z** | | **p** | |
| Trait (I-EC) | | |  | → |  | | Education score | | | |  | | 0.252 | |  | | 0.1067 | |  | 2.36 |  | 0.018 |  |
| Education score | | |  | → |  | | State EC | | | |  | | 0.245 | |  | | 0.0716 | |  | 3.41 |  | < .001 |  |
| Trait (I-EC) | | |  | → |  | | State EC | | | |  | | 0.374 | |  | | 0.0872 | |  | 4.29 |  | < .001 |  |
|  | | | | | | | | | | | | | | | | | | | | | | | |

# References

1. Wagenmakers, E.-J. *et al.* Bayesian inference for psychology. Part II: Example applications with JASP. *Psychon. Bull. Rev.* **25**, 58–76 (2018).
